# Mechanisms Underlying Cognitive Effects of Inducing a Mindful State

**DOI:** 10.5334/joc.205

**Published:** 2022-01-21

**Authors:** Elena Vieth, Lisa von Stockhausen

**Affiliations:** 1 Department of Psychology, University of Duisburg-Essen – Campus Essen, Essen, DE

**Keywords:** mindfulness induction, relaxation, attentional networks, updating/working memory, cognitive inhibition, task switching

## Abstract

Mindfulness is understood as a state or practice of guiding attention to the present moment without judgment. While some studies on mindfulness-based interventions demonstrate beneficial effects on cognitive functions (e.g. Chiesa et al., 2011; Yakobi et al., 2021) it still appears challenging to identify underlying mechanisms due to the wide range of research designs and dependent measures used, as well as the frequent absence of active control conditions. Relatedly, processes underlying the effects of short inductions of a mindful state may be unspecific to mindfulness and attainable through other means, such as relaxation (Fell et al., 2010).

Therefore, the current study compared the effects of a brief mindfulness induction with a relaxation induction (via progressive muscle relaxation; active control condition) and listening to podcasts (passive control condition) in a pre-post experimental design. 78 participants without recent meditation experience were randomly assigned to the experimental conditions (mindfulness = 25; progressive muscle relaxation = 24; podcast listening = 30) and received corresponding instructions for a total of 40 minutes (2 × 20 minutes) a maximum of 3 days apart. Executive functions of inhibition, updating and switching as well as attentional networks were assessed with the continuous performance task, n-back task, number-letter task, and attention network task, respectively.

While updating and executive attention similarly benefited from meditation and relaxation compared to podcast listening, inhibition and shifting measures indicate differential effects of mindfulness induction. Alerting and orienting were not affected by any induction. Implications for mechanisms underlying the effects of mindfulness are discussed.

There is a fast-growing literature on the potential effects of short inductions of a mindful state and mindfulness-based interventions on attention and executive functions (Chiesa et al., 2011; Guendelman et al., 2017; Tang et al., 2015; Yakobi et al., 2021). A common practice during these interventions is breathing meditation, during which participants are asked to guide their attention to the natural flow of their breathing and observe any internal events that may arise (such as thoughts, perceptions, or emotions) without engaging with or judging them. If the mind wanders off, practitioners are to let go of all distractions and return their attention to their breath. This task can be challenging, as our minds are easily distracted by internal and external events that attract our attention. Such breathing meditation practice reflects what is considered the essence of mindfulness in contemporary scientific approaches, as expressed by Kabat-Zinn (1994, p. 4), for example: *Mindfulness means paying attention in a particular way: on purpose, in the present moment, and non-judgmentally*.

Several models of mindfulness that specify components of a mindful state and underlying mechanisms have been proposed (e.g., Bishop et al., 2004; Hölzel et al., 2011; Malinowski, 2013; Shapiro et al., 2006). All of these models include attention regulation as a component, as there is consensus that attention regulation and executive control are required for guiding and maintaining attentional focus on a task within any meditation practice. Bishop et al.’s two-component model of mindfulness defines mindfulness as a meta-cognitive skill comprising attention regulation, in the sense of monitoring one’s attention to the object of focus (i.e. the breath), and executive control, in the sense of switching one’s attention back to the breath when distractions occur and inhibiting the elaborative processing of thoughts, feelings and sensations while maintaining an open and accepting attitude towards experience. Malinowski’s (2013) Liverpool Mindfulness Model is based on the network model of attention by Posner and Peterson (1990; Peterson & Posner, 2012), which comprises an alerting network, an orienting network and a network for executive attention. During meditation, these networks are engaged to sustain attention on a selected object (such as the breath; alerting network), to disengage from mind wandering (executive attention network) and to shift attention back to the task (orienting network). Consequently, Malinowski considers attention to be a core feature of a mindful state. Shapiro et al.’s model (2006) comprises attention, intention and attitudes as central components whose interplay induces mindfulness on a moment-to-moment basis, namely through the experience of paying attention with a kind and open attitude, rooted in intentions about why one practices. Hölzel et al. (2011) propose attention regulation as one component mechanism of mindfulness alongside improved body awareness, emotion regulation and a changed perspective on the self. Thus, all of the described models consider improved attentional processes by way of improved sustained attention and better monitoring as well as more effective executive functioning (e.g., inhibiting irrelevant content, shifting between task sets; see Suchy, 2009; Miyake et al., 2000) as possible mechanisms contributing to the effects of mindfulness training. Furthermore, Jha et al. (2019) have argued that since aspects of attentional control (such as dis-/engagement, maintenance and monitoring) are essential for successful maintenance and manipulation of information held in working memory, improvements in attentional control following mindfulness trainings may be beneficial for working memory as well. However, any further specification of the mechanisms underlying mindfulness practice requires a more detailed consideration of what exactly is being practiced. In order to systematize frequently studied forms of practice, Lutz and colleagues (2008) proposed the now widely accepted distinction between focused attention meditation (FAM; i.e. sustained focus on a selected object, such as the breath) and open monitoring meditation (OMM; i.e. constantly monitoring whatever is experienced without focusing on and reacting to any particular object or process). FAM should narrow attentional focus and strengthen top-down attentional control whereas OMM should widen attentional focus and reduce attentional control (Lippelt et al., 2014; for empirical results see below).

In order to specify mechanisms through which mindfulness practice affects attention and executive functioning, it seems further necessary to separate the effects of inducing a mindful state (through one meditation of 10 to 30 minutes in length) from the effects of repeated practice (i.e. brief mindfulness trainings) or even sustained practice over weeks or years (i.e. mindfulness interventions or mindfulness-based therapies; Heppner & Shirk, 2018). Any measurable effect of a mindfulness induction necessarily represents a transient state which may only stabilize with repeated practice and can be described as the starting point for longer-lasting changes in cognitive functions. However, it is not yet clear what exactly this transient starting point might be.

Different proposals have been brought forward as to how short mindfulness inductions affect performance in tasks requiring attention and executive control. Studies by Wenk-Sormaz (2005; employing a Stroop task, among others) and Ostafin and Kassman (2012; studying insight problem-solving) suggest that FAM inductions reduce automatized cognitive processing by means of improved inhibition or enhanced retrieval of non-habitual information due to improved set shifting and higher cognitive flexibility (Suchy, 2009). Furthermore, Colzato et al. (2015, 2016) investigated differential effects of one-time FAM and OMM and conclude that OMM results in more parallel attention allocation – reducing attentional blinks in a rapid serial presentation task, for example – but also increases likelihood of responding to irrelevant stimulus features. By contrast, FAM increases top-down control, leading to better inhibition of irrelevant stimulus features.

In contrast to these proposed specific effects of single meditations, it has been argued that early stages of various meditational practices are characterized by processes unspecific to meditation, such as relaxation. For example, Fell et al. (2010) argue that during their first attempts to meditate, people may not succeed in keeping their attention focused. After an initial habituation phase, most meditation practices result in greater calmness and relaxation, which, however, could also be achieved with relaxation techniques. Only advanced practitioners may undergo meditation-specific processes and accordingly attain meditation-specific changes. The authors present evidence from EEG recordings of practitioners with different levels of expertise and show that certain changes during early stages of meditation practice, such as increased power and synchronization in alpha and theta activity, do not depend on the technique or on expertise. By contrast, other changes, such as increased power and synchronization in gamma band activity, depend on expertise and represent longer-lasting structural changes. In light of Fell and colleagues’ findings of unspecific changes in early stages of meditational practice, it therefore appears to be possible that short mindfulness inductions do not directly improve attention and executive control, but are helpful because they reduce dysfunctional tension and free up resources for improved cognitive performance, for example. The findings by Colzato et al. (2015; 2016) reported above may suggest otherwise, but even in their studies differences between FAM and OMM were not found for all expected measures (see also Colzato, Sellaro, Samara & Hommel, 2015). Relatedly, Ainsworth et al. (2013) did not find differential effects of a brief mindfulness training with FAM vs. OMM on executive control (measured with a variant of the attention network task; ANT). Thus, if differences between FAM and OMM cannot be consistently found in studies of short inductions or brief trainings, this may suggest that effects at this stage of practice are unspecific to mindfulness meditation and that evidence so far does not eliminate the possibility of relaxation contributing to the cognitive effects of short mindfulness inductions.

Studies seeking to compare meditation and relaxation do not provide conclusive evidence of differential effects either, partly because several such studies using rest or relaxation as control conditions did not provide participants with instructions on how to obtain a state of rest or relaxation. Therefore, it is unclear whether or to what degree the participants actually achieved relaxation, influencing the studies’ implications. In Wenk-Sormaz’s study (2005), for example, the rest group was told to sit, rest, let their minds wander and stay awake. Mrazek et al. (2012, Experiment 2) showed that an eight-minute meditation reduced participants’ errors and reaction time variability on a Go/No-Go task compared to a passive rest condition in which the rest group was instructed to relax and not fall asleep. The following two studies gave more detailed instructions to the relaxation group. Comparing the effects of a short meditation practice using integrative body-mind training (IBMT, which comprises relaxation, breathing practice and mental imagery) versus a relaxation training, Tang et al. (2007) found no group differences on the ANT for alerting or orienting, but higher scores in the IBMT group for executive attention. While these findings suggest that a combined meditation and relaxation training is more beneficial for executive attention than a pure relaxation training, it is difficult to specify the impact of meditation alone. Johnson et al. (2015) compared the effects of a single mindful breathing meditation with a sham meditation (instructions to breathe deeply, relax and to sit still in silence) and an audiobook control group. In their post-test-only design, both meditation and sham meditation exhibited positive effects on state mindfulness and mood compared to the control group, but no effects on attention or working memory were found. To summarize, based on the existing evidence, it cannot be ruled out that the cognitive benefits of brief mindfulness inductions are caused by non-meditation-specific states and that relaxation is a main component of this process. Investigating the specific effects of a mindfulness induction as compared to relaxation requires clearly separating mindfulness from relaxation and providing instructions for both states in comparable detail. It also requires a randomized pre-/post design with an active and passive control condition to control for both effects unspecific to mindfulness practice as well as effects of repeated testing. This research gap is addressed in the present study.

In a randomized controlled trial with an active and passive control group, we compared the effects of a short mindfulness induction (2 × 20 minutes of breathing meditation instructions) to the effects of a progressive muscle relaxation technique (PMR) training of the same length as an active control condition and podcast listening as a passive control condition to control for the effects of repeated testing. This design allows us to identify possible specific effects of mindfulness induction beyond mere relaxation on attention and executive control. Breathing meditation was selected as an effective form of focused attention practice for beginners. Completing two sessions of 20 minutes allowed participants in the mindfulness condition to become more familiar with the practice of controlling their attentional focus on the breath than is possible in a one-time trial, while still sufficiently restricting exposure to training such that no long-term processes specific to mindfulness could unfold. PMR was selected as a standardized and evidence-based set of instructions for achieving a relaxed state (Manzoni et al., 2008; McCallie et al., 2006). Testing for effects after such short-term inductions aims at identifying processes during the first stages of mindfulness intervention, before longer-term processes can unfold through practice. We assessed the fundamental executive functions of set formation, set maintenance, and set shifting (Miyake et al., 2000; Suchy, 2009) and employed the ANT (Fan et al., 2002) to assess attentional networks (Petersen & Posner, 2012; Posner & Petersen, 1990). If the induction of a mindful state in particular improves attention and executive control, it was hypothesized that the mindfulness group should outperform both PMR and the passive control group. If relaxation is the essential effect in short inductions, both mindfulness and PMR should outperform the passive control group.

## Methods

### Participants

Seventy-nine participants who were recruited on campus and through Facebook groups took part in the experiment. The sample was a white European sample (68 female) and ranged in age from 18 to 65 years (*M* = 26,44, *SD* = 10,2). Based on a screening questionnaire, individuals who were younger than 17 and/or reported having engaged in meditation or other mindfulness practices during the last three months were excluded from the study. To ensure accurate measurements for the questionnaires and comprehension of instructions, German was required to be participants’ first language. Persons who met all criteria were contacted by e-mail or telephone and invited to participate in two experimental sessions in a laboratory at the University of Duisburg-Essen. They were provided with written information about the methodology used, data protection and research ethics prior to participation. After completing the study, participants received either course credit or a booklet and CD containing information about mindfulness meditation and instructions for guided meditations for compensation. Data storage and anonymization met the standards of the European Union’s General Data Protection Regulation (GDPR 2016/679). The experiment was approved by the ethical commission of the Department of Psychology at the University of Duisburg-Essen, Germany (Ethics Vote 2019/26/07).

### Assessment of Executive Functions and Attention Networks

All tasks were programmed with and presented (including standardized written instructions) via the Experiment Builder software (SR Research Ltd., 2015). Participants viewed the stimuli on a 23-inch Dell LED monitor from a distance of approximately 50 cm. Responses were recorded with a Cedrus RB-540 response pad (Cedrus Corporation, 2019). In all tasks, participants were instructed to respond as quickly as possible without making mistakes.

#### Continuous Performance Test-II

**Figure 1:**
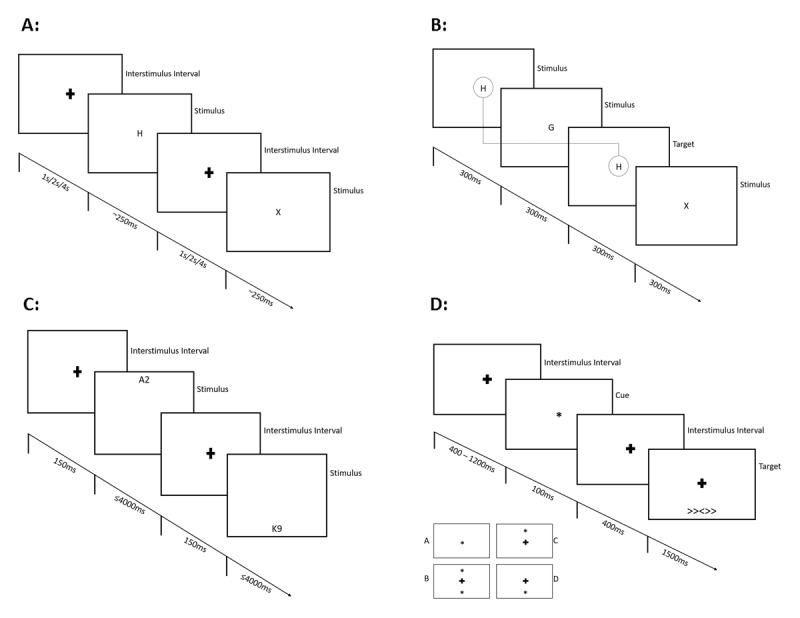
Display Sequences of the Reaction Time Tasks. *Note*: Panel A: During the CPT-II, participants were presented with a consecutive stream of single capital letters (for example: H – O – T – X – Z) and were to press a button every time a letter appeared on the screen, except for the letter “X” (90% target trials; 10% non-target trials). Panel B: During the N-Back Task, in a 2-back block (n = 2), participants were to indicate a target trial if the following stream of letters occurred: H – G – H, but not if the presented letters were: H – G – X. Panel C: During the Number-Letter Task, if the number-letter combination appears in the upper half of the screen, participants are instructed to indicate whether the presented number is odd or even. If the number-letter combination appears in the lower half of the screen, participants are asked to indicate whether the letter is a consonant or a vowel. Panel D: During the Attention Network Task, in no cue trials, participants were presented with a fixation cross in the center of the screen prior to target onset. In center cue trials (A), an asterisk was shown in the center of the screen instead of a fixation cross. In double cue trials (B), two asterisks were presented simultaneously at the two possible target locations (above and below the fixation cross). In spatial cue trials (C and D), the cue was displayed at the position of the upcoming target.

The Continuous Performance Test-II (also called non-X CPT; Conners et al., 2003; Conners, 2004) is a variant of the CPT used to investigate participants’ capacities for set maintenance/cognitive inhibition (Suchy, 2009) by assessing executive control and impulse control in response to a rarely occurring non-target. Participants were presented with consecutive letters and had to press a button every time a letter other than the letter “X” appeared on the screen (90% target trials; 10% non-target trials). Reaction times for correct and incorrect responses as well as frequencies of correct and incorrect responses were collected to assess participants’ ability to react to target trials while inhibiting a response to distractor trials.^1^ Inclusion of different interstimulus intervals (ISIs; 1000 ms, 2000 ms, 4000 ms) allowed us to investigate participants’ ability to adapt to task demands (Ballard, 2001). ***Figure 1*** shows a display sequence with presentation durations and ISIs. Participants were instructed to place their right index finger on the response button and to indicate targets by pressing the button. During the task, participants wore headphones for noise cancellation. A session consisted of 360 trials separated into 18 experimental blocks (20 trials per block). After three blocks, participants were instructed to take a break. Total task duration was 14 minutes on average.

#### N-Back Task

The N-back task (Kirchner, 1958) is used to assess set formation and working memory capacity (Chatham et al., 2011; Suchy, 2009). Participants are presented with a stream of individual letters and are asked to indicate whether the current letter matches the one shown *n* steps before. To complete the task, participants need to keep information about previous stimuli in memory, make a comparison with the current stimulus, and constantly update the information held in memory. The factor *n* is varied between blocks to increase or decrease the task’s difficulty (see ***Figure 1*** for an example from a 2-back block). Reaction times of correct and incorrect responses as well as frequencies of correct and incorrect responses were collected to assess participants’ ability to react to n-back and non-back trials. Participants were instructed to place their respective index fingers on a left and right button on the response pad and press these buttons to indicate n-back and non-n-back trials respectively. During the task, participants wore headphones for auditory feedback during the practice blocks and for noise cancellation during the remaining tasks. A session consisted of two practice and eight experimental blocks (two 0-, 1-, 2- and 3-back blocks each in randomized order); each experimental block contained 48 trials, following which participants were instructed to take a break. Total task duration was 25 minutes on average.

#### Number-Letter Task

The Number-Letter Task assesses the cognitive ability of set shifting/task switching (Rogers & Monsell, 1995; Suchy, 2009). Participants are presented with pairs of numbers and letters (for example A2, K9) above or below a fixation cross. If the number-letter pair appears in the upper half of the screen, participants must indicate whether the number is odd or even. If the pair appears in the lower half of the screen, participants must indicate whether the letter is a consonant or a vowel. Stimuli occurred equally often in both positions, in randomized order, resulting in switch trials when the stimulus position (and thus also the task) changed and non-switch trials when the stimulus position remained the same. Participants’ reaction times for correct and incorrect responses as well as frequencies of correct and incorrect responses in non-switch and switch trials were recorded to assess participants’ ability to flexibly switch between tasks (***Figure 1*** shows a display sequence). Participants were instructed to place their respective index fingers on left and right buttons on the response pad, which they then used to specify whether the number was odd or even or the letter was a consonant or vowel, depending on the task. During the task, participants wore headphones for noise cancellation. A session consisted of three short practice blocks and one experimental block. Participants received feedback during the practice blocks. The experimental block contained 160 trials and was presented in a single run. Total task duration was about 17 minutes.

#### Attention Network Task

The ANT assesses the networks of alerting, orienting and executive attention based on the model of attention by Posner and Petersen (Fan et al., 2002; Petersen & Posner, 2012; Posner & Petersen, 1990). Different conditions within the task allow the efficacy of the three networks to be assessed separately. The ANT used in this experiment was developed and evaluated by Weaver et al. (2013). We recorded reaction times for correct and incorrect responses as well as frequencies of correct and incorrect responses to assess participants’ alerting, orienting and executive networks. Participants were presented with a left- or right-facing arrow (target) in the upper or lower half of the screen and were asked to indicate the direction of the target by pressing a button. The target was flanked by two arrows on each side, which either pointed in the same (congruent) or opposite (incongruent) direction of the target arrow. RTs and frequencies of responses for congruent and incongruent flanker trials were used to calculate the effect of the executive network (Executive = incongruent – congruent; Fan et al., 2002).

In some trial conditions, participants were presented with a brief cue prior to the onset of the target. ***Figure 1*** shows the sequence of a cue and target presentation and the four different cue conditions. In *no cue* trials, no cue appeared; therefore, neither the alerting nor orienting network was expected to be activated. *Double cues* were presented simultaneously at the two possible target locations and expected to engage the alerting network by forewarning the participant of the upcoming target at each target location. Comparing response times and response accuracy between these two cue conditions therefore allowed us to calculate the effect of the alerting network (Alerting = double cue – no cue). *Spatial cues* occurred at the same position as the upcoming target and should activate both the alerting and orienting networks for the subsequent target presentation. *Center cues* were shown in the center of the screen and used to alert participants of the upcoming target, without providing any orientation as to possible target locations. Comparing response times and response accuracy between these two cue conditions allowed us to calculate the effect of the orienting network (Orienting = spatial cue – center cue).

Participants were instructed to place their left and right index fingers on respective buttons on the response pad and to indicate the direction of the target stimulus for each trial. Participants wore headphones for noise cancellation. A session consisted of a practice block (24 trials) and three experimental blocks with 96 trials each. Each experimental block included all 4 cue conditions in randomized order. The practice block lasted up to 2 minutes, while each experimental block took approximately 5 minutes.

### Questionnaires

To control for possible influences of mood on attention and executive control (Van Steenbergen et al., 2010) and for a priori differences in dispositional mindfulness, the Positive and Negative Affect Schedule (PANAS, Watson et al., 1988; German: Breyer & Bluemke, 2016) and the Mindful Attention and Awareness Scale (MAAS, Brown & Ryan, 2003; German: Michalak et al. 2008) were employed. The scales and their respective results are described in detail in Appendix A.

### Procedure

The experiment consisted of two sessions. Participants were greeted by the experimenter and given the written informed consent form. After participants read the form, asked questions and gave their consent, the first session started with the CPT, followed by the N-back task, MAAS, PANAS, number-letter task, and ANT. This sequence was identical for all participants. After the pre-measurement, participants received their first practice session. Depending on the experimental condition, they either received oral instructions (mindfulness/PMR) or listened to a randomly selected podcast with headphones. Afterwards, participants were thanked and invited to participate in the second session. The two sessions were a maximum of three days apart. Participants did not engage in practice between the sessions. The session started with the second mindfulness or PMR practice or listening to a different podcast. Afterwards, participants again completed all tasks and questionnaires. At the end, they received their reward for participation and were thanked by the experimenter.

### Research Design

The research design was a 3 (mindfulness meditation, progressive muscle relaxation or podcast listening) × 2 (time of measurement) experimental design. Participants were randomly assigned to one condition, and measurements took place pre- and post-induction or podcast listening. While mindfulness meditation served as the experimental condition, progressive muscle relaxation was the active control group and listening to podcasts served as a non-treatment control condition. Random assignment was ensured via a randomized number list created by the first author, according to which participants were assigned to either the induction of a mindful state (n = 25), relaxation (n = 24) or the podcast listening condition (n = 30) by the experimenter at the beginning of their first session. Participants were not aware of their assignment or the existence of different conditions.

### Experimental Conditions

The mindfulness and relaxation inductions as well as podcast listening each took approximately 20 minutes and were conducted twice with a maximum of three days in between. This procedure allowed participants in the mindfulness condition to become more familiar with the practice of controlling their attentional focus than is possible in a one-time trial, while still restricting practice sufficiently that no long-term processes specific to mindfulness could unfold. Instructions (see Appendix B) were read to participants by the experimenter, who had been trained by the authors to deliver both mindfulness meditation and PMR inductions from a written script with a calm voice. To ensure clear differentiation between the mindfulness and relaxation conditions, the mindfulness instructions did not contain any phrases implying or directly instructing participants to engage in relaxation. Instructions for the progressive muscle relaxation were based on Jacobson (1938) and adjusted so that they did not include mindfulness-related phrasing (e.g. accepting the present experience). Podcasts were pretested for not evoking strong emotional reactions (assessed via levels of subjective arousal and valence) and for eliciting an average level of interest and engagement. The three selected podcasts concerned historical sites and exceptional landscapes.

### Data Analysis

Generalized linear mixed models (GLMM) were used to analyze the reaction time data. GLMMs allow for the analysis of single-trial, raw RT data without applying (non-linear) transformations or averaging across participants. In doing so, we accounted for the typically positively skewed distribution of empirical reaction times (Balota & Yap, 2011; Lo & Andrews, 2015) and for meaningful differences in response patterns within and between individuals (Speelman & McGann, 2013). Additionally, the effect structure of GLMMs makes it possible to specify multiple sources of non-independence within the data (Brauer & Curtin, 2018). For theoretical reasons and based on the model fit for the current data compared to other functions, the inverse Gaussian distribution (Johnson et al., 1970; Tweedie, 1957) was selected. In order to account for possible influences of accuracy on response times, we included the accuracy factor in our models for the RT analysis.

The signal detection measure of discriminability (*d’* = z[Hits] – z[False Alarms]) was analyzed with linear regressions. Response frequencies were analyzed with multilevel logistic regressions, which allow for the modelling of a binomial distribution while taking data dependencies into account. Contrast coding schemes for accuracy models are equal to the respective generalized linear mixed model.

In addition to the task-specific fixed effects of interest, experimental condition and time of measurement (pre/post) were included as a fixed-effect interaction term. Where applicable, we additionally added a three-way interaction including task-specific factors of interest. As recommended by Barr et al. (2013), we included random slopes for the highest-order combination of within-unit factors included in the interactions. The models’ significance was tested via likelihood ratio chi-square tests (with maximum likelihood estimation) in which the full model was compared to restricted models and a null model. Since the interactions included in the models compare either mindfulness or PMR with the reference category of podcast listening (treatment coding scheme), we utilized planned comparisons of the respective interactions’ estimated marginal means for follow-up analyses comparing pre-/post differences between all three conditions and also between test-specific factor levels affecting dependent variables (such as ISI for the CPT-II or n-level for the n-back task) included in the three-way interactions. We utilized the Tukey method for comparing a family of 3 estimates to control for heightened Type I error when carrying out multiple comparisons. Further information on model building, contrast coding for each test, the statistical software utilized as well as full models including 95% confidence intervals can be found in Appendix C.

All data files are available on the Open Science Framework (DOI 10.17605/OSF.IO/QN784).

## Results

The RT data were cleaned for RTs below 100 ms and above 1500 ms. Unless otherwise specified, the RT data included both correct and incorrect trials, allowing for the modelling of accuracy as a fixed effect. The cut-off value for excluding participants after data cleaning was more than 40% of trials missing. With respect to accuracy, we examined the data for respondents with low performance (share of correct trials < 50%). No participant needed to be excluded based on this criterion.

The description of results will focus on the hypotheses examined in this paper (i.e. interactions with time of measurement and condition and associated simple main effects) and task-specific effects of interest. Only effects with a significance level of p ≤ 0.05 are reported.

### Continuous Performance Task

One participant was removed due to too many missing data points (only 2 data points were available; total data loss: 0.07%).

#### Reaction Time

As incorrect responses (i.e. responses to non-targets) were rare, only correct responses were included in the analysis. The model included a fixed effect for age, a random slope for time of measurement by participant and a three-way interaction between time of measurement, condition, and ISI (with 1000 ms as the reference category), and thus also all two-way interactions containing these factors (see full model in Appendix C, Table C1). ISI was included in the three-way interaction since specific effects of mindfulness in the sense of improved cognitive inhibition might be particularly likely to surface within shorter ISIs.^2^

##### Simple and Main Effects

The analysis showed a main effect of age, *β* = 2.13, *SE* = 0.42, *p* < 0.001, with RT increasing as age increased. Simple effects were present for ISI 2000 ms, *β* = 15.26, *SE* = 0.80, *p* < 0.001, and 4000 ms, *β* = 49.13, *SE* = 0.88, *p* < 0.001, indicating slower RTs for longer ISIs, and for time of measurement, *β* = –11.74, *SE* = 2.91, *p* < 0.001, with RTs decreasing from pre- to post-measurement.

##### Interactions of Interest

A two-way interaction between time of measurement and condition (mindfulness) was found, *β* = –15.04, *SE* = 2.80, *p* < 0.001, with a larger decrease in RT from pre- to post measurement for mindfulness compared to podcast listening. A two-way interaction between time of measurement and ISI (2000 ms) was found, *β* = 6.98, *SE* = 1.47, p < 0.001, suggesting less pre-post improvement in RT for an ISI of 2000 ms compared to an ISI of 1000 ms. All other two-way interactions including the factor time of measurement were non-significant, *p* ≥ 0.527. There was also a three-way interaction between time of measurement, condition, and ISI for mindfulness and the ISI of 2000 ms, *β* = 8.04, *SE* = 2.23, *p* < 0.001, indicating that in the mindfulness compared to the podcast condition, RTs decreased more strongly from pre- to post-measurement for the shorter ISI of 1000 ms compared to 2000 ms. No significant difference was found for mindfulness and the ISI 4000 ms compared to 1000 ms, *p* = 0.106. The three-way interaction between PMR, time of measurement and ISI was significant for both ISI of 2000 ms, *β* = –17.11, *SE* = 2.57, *p* < 0.001, and ISI of 4000 ms, *β* = –21.63, *SE* = 2.83, *p* < 0.001, indicating that after PMR (compared to listening to podcasts), RTs decreased more strongly from pre- to post-measurement for both the ISI of 2000 ms and 4000 ms compared to the ISI of 1000 ms.

Likelihood ratio tests compared the model fit to a set of restricted models (see Appendix D). The described model fit significantly better than a model with a two-way interaction of time of measurement by condition only, χ*²* (10) = 106.99, *p* < 0.001, a model without interaction terms, χ² (12) = 110.13, *p* < 0.001, and a null model, χ² (18) = 3134.6, *p* < 0.001.

**Figure 2:**
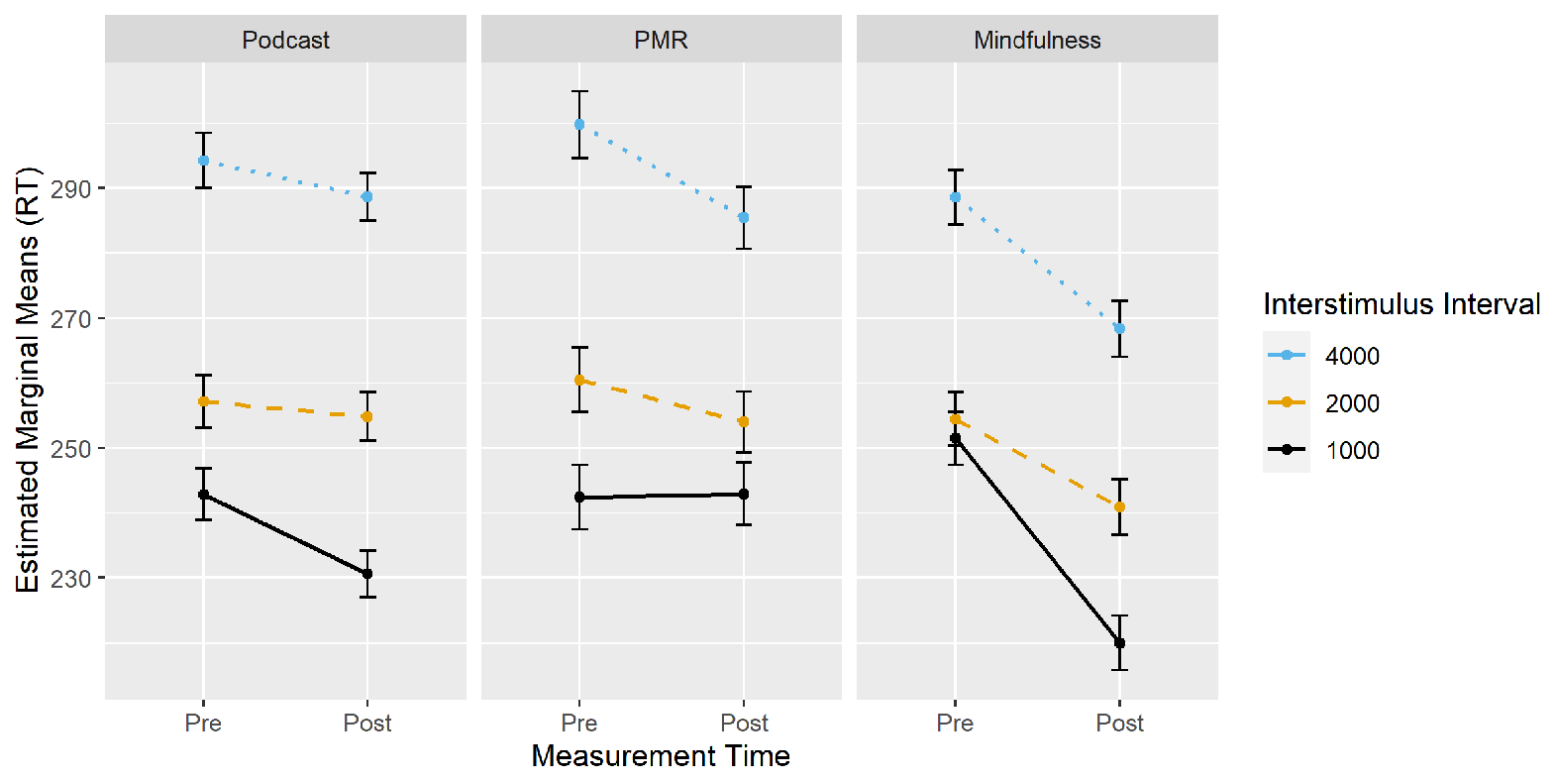
CPT-II: Changes in RT from Pre- to Post-Measurement by Condition and ISI.

***Figure 2*** displays the EMMs for the three-way interaction between time of measurement, condition and ISI. Planned comparisons were computed with RT change scores (EMM_t1_ – EMM_t0_) between all conditions within each ISI (see ***Table 1***). For the ISI of 1000 ms, the increase in speed from pre- to post-measurement was larger after mindfulness induction than after podcast listening, while the increase in speed was smaller after PMR than after podcast listening as well as after mindfulness. For the ISI of 2000 ms, the increase in speed was larger after mindfulness than after podcast listening. For the ISI of 4000 ms, both induction groups exhibited a larger increase in speed from pre- to post-measurement than the podcast listening group.

**Table 1:** Planned Comparisons of Measures of Attention and Executive Control for Significant Interactions in Generalized Linear Mixed Models and Regression Analyzes.


	PODCAST – MINDFULNESS			PODCAST – PMR			MINDFULNESS – PMR	
		
	*T1 – T0: ESTIMATE (SE)*	*p*		*T1 – T0: ESTIMATE (SE)*	*p*		*T1 – T0: ESTIMATE (SE)*	*p*

CPT–II RT (ISI_1000_)	19.22 (2.95)	**<.001**		–12.86 (3.07)	**<.001**		–32.08 (4.33)	**<.001**

CPT–II RT (ISI_2000_)	11.18 (3.36)	**0.003**		4.25 (3.39)	0.423		–6.93 (4.43)	0.260

CPT–II RT (ISI_4000_)	14.72 (3.43)	**<.001**		8.77 (3.73)	**0.049**		–5.95 (4.61)	0.400

CPT–II *d’*	–0.30 (0.25)	0.453		–0.62 (0.24)	**0.028**		–0.32 (0.24)	0.386

CPT–II Errors of Omission	0.04 (2.85)	1.000		6.64 (2.75)	**0.043**		6.60 (2.78)	**0.048**

N–Back RT	36.00 (6.70)	**<.001**		21.20 (5.02)	**<.001**		–14.70 (7.20)	0.101

N–Back *d’*	–	–		–	–		–	–

N–Back Errors of Omission	–1.32 (1.01)	0.396		–2.06 (1.02)	0.108		–0.74 (1.05)	0.762

Number–Letter RT	2.76 (6.68)	0.910		–29.40 (7.73)	**<0.001**		–32.16 (9.75)	**0.003**

Number–Letter Accuracy	–	–		–	–		–	–

ANT Executive Network RT	12.66 (2.58)	**<.001**		14.02 (2.57)	**<.001**		1.36 (3.33)	0.913

ANT Executive Network Accuracy	–	–		–	–		–	–

ANT Alerting Network RT	–3.51 (4.27)	0.690		–9.54 (4.39)	0.076		–6.03 (4.23)	0.327

ANT Alerting Network Accuracy	–	–		–	–		–	–

ANT Orienting Network RT	3.13 (3.78)	0.686		–6.49 (4.54)	0.326		–9.61 (4.93)	0.125

ANT Orienting Network Accuracy	–	–		–	–		–	–


*Note*: T0 = pretest; T1 = posttest. P value adjustment: Tukey method for comparing a family of 3 estimates. Empty rows represent models that did not produce any significant effects of interest but are reported in the Results section.

Taken together, the results indicate that both induction conditions resulted in RT benefits compared to the podcast listening condition, although in different ways. The RT benefit of mindfulness was already apparent at the shortest ISI, whereas the benefit of PMR only arose at longer ISIs. Interestingly, for the short ISI, performance declined after PMR compared to the other two groups.

#### Accuracy Analysis

Discriminability (*d*’) was analyzed with a model including a fixed effect for age and a three-way interaction between time of measurement, condition and ISI (see full model in Appendix C, Table C2). The analysis yielded a simple effect for ISI, 4000 ms, *β* = 0.39, *SE* = 0.12, *p* = 0.001, indicating better discriminability following an ISI of 4000 ms compared to 1000 ms. All other simple and main effects were non-significant, p ≥ 0.227. Moreover, a two-way interaction between time of measurement and condition (PMR) was found, *β* = 0.62, *SE* = 0.24, *p* = 0.010, with a greater increase in *d’* from pre- to post measurement for PMR compared to podcast listening. All other two-way interactions including the factor time of measurement were non-significant, *p* ≥ 0.230. All three-way interactions were also non-significant, *p* ≥ 0.260.

**Figure 3:**
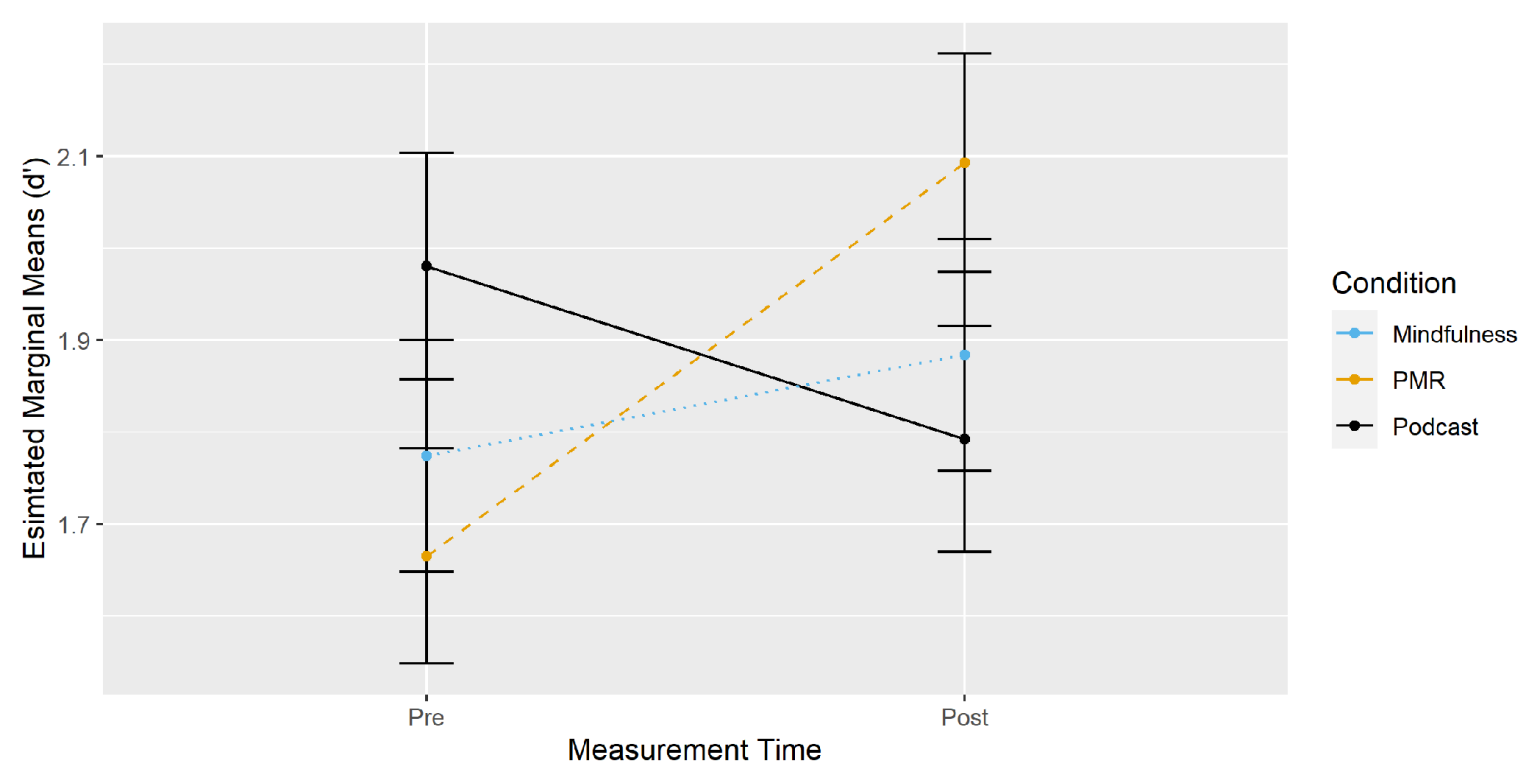
CPT-II: Changes in d’ from Pre- to Post-Measurement by Condition.

***Figure 3*** displays EMMS for the two-way interaction of time of measurement by condition. Planned comparisons were computed with *d’* change scores (EMM_t1_ – EMM_t0_, see ***Table 1***). As already suggested by the two-way interaction, the increase in *d’* was higher after PMR induction than after podcast listening.

To further differentiate the processes underlying performance, analogous models were run with errors of omission (i.e. misses) as the dependent measure (see Appendix C, Table C3). They showed a two-way interaction between time of measurement and condition (PMR), *β* = –6.64, *SE* = 2.75, *p* = 0.016, indicating a greater reduction in errors of omission from pre- to post-measurement for PMR compared to the podcast listening group; all other interactions including the factor time of measurement were non-significant, *p* ≥ 0.227. EMM contrasts (***Table 1***) showed a lower number of misses after PMR compared to both podcast listening and mindfulness.

The results indicate that inducing relaxation through PMR increased discriminability and reduced errors of omission compared to both mindfulness and podcast listening. Mindfulness did not affect discriminability compared to listening to a podcast.

### N-back Task

One participant was removed due to too many missing data points (data loss through data cleaning: 6.59%).

#### Reaction Time

The model included a fixed effect for target, age and accuracy, a random slope for time of measurement by participant and a three-way interaction of time of measurement by condition by n-back level (including 1-, 2- and 3-back trials; 1-back as the reference category; see Appendix C, Table C4). N-back level was included in the three-way analysis because possible specific effects of mindfulness induction might be particularly likely to surface in the more difficult n-conditions which require more working memory engagement.^3^

##### Simple and Main Effects

The analysis showed a main effect for accuracy, *β* = –9.41, *SE* = 2.59, *p* < 0.001, with shorter RT for accurate compared to inaccurate trials, and a main effect for target type, *β* = 8.88, *SE* = 1.24, *p* < 0.001, indicating longer RTs for target compared to non-target trials. Simple effects were present for the n-back levels 2-back, *β* = 68.47, *SE* = 1.81, *p* < 0.001, and 3-back, *β* = 85.23, *SE* = 1.84, *p* < 0.001, indicating longer RTs for higher n-trials, and for time of measurement, *β* = –74.73, *SE* = 3.51, *p* < 0.001, with a decrease in RT from pre- to post-measurement.

##### Interactions of Interest

There was a two-way interaction between time of measurement and condition: mindfulness, *β* = –35.98, *SE* = 6.70, *p* < 0.001, and PMR, *β* = –21.24, *SE* = 5.02, *p* = 0.002, with a larger decrease in RT for both mindfulness and PMR compared to podcast listening from pre- to post-measurement, and for time by n-back: 2-back, *β* = –27.38, *SE* = 2.58, *p* < 0.001, and 3-back, *β* = –10.85, *SE* = 2.77, *p* = 0.008, indicating a larger decrease in RT from pre- to post measurement for 2-back and 3-back trials compared to 1-back trials. The analysis showed no significant three-way interaction between time of measurement, condition, and n-back, *p* ≥ 0.150.

Likelihood ratio tests (cf. Appendix D) showed that the described model fit significantly better than a model with a two-way interaction of time of measurement by condition only, χ² (10) = 83.64, *p* < 0.001, a model with no interaction terms, χ² (12) = 85.76, *p* < 0.001, and a null model, χ² (20) = 2257.90, *p* < 0.001.

**Figure 4:**
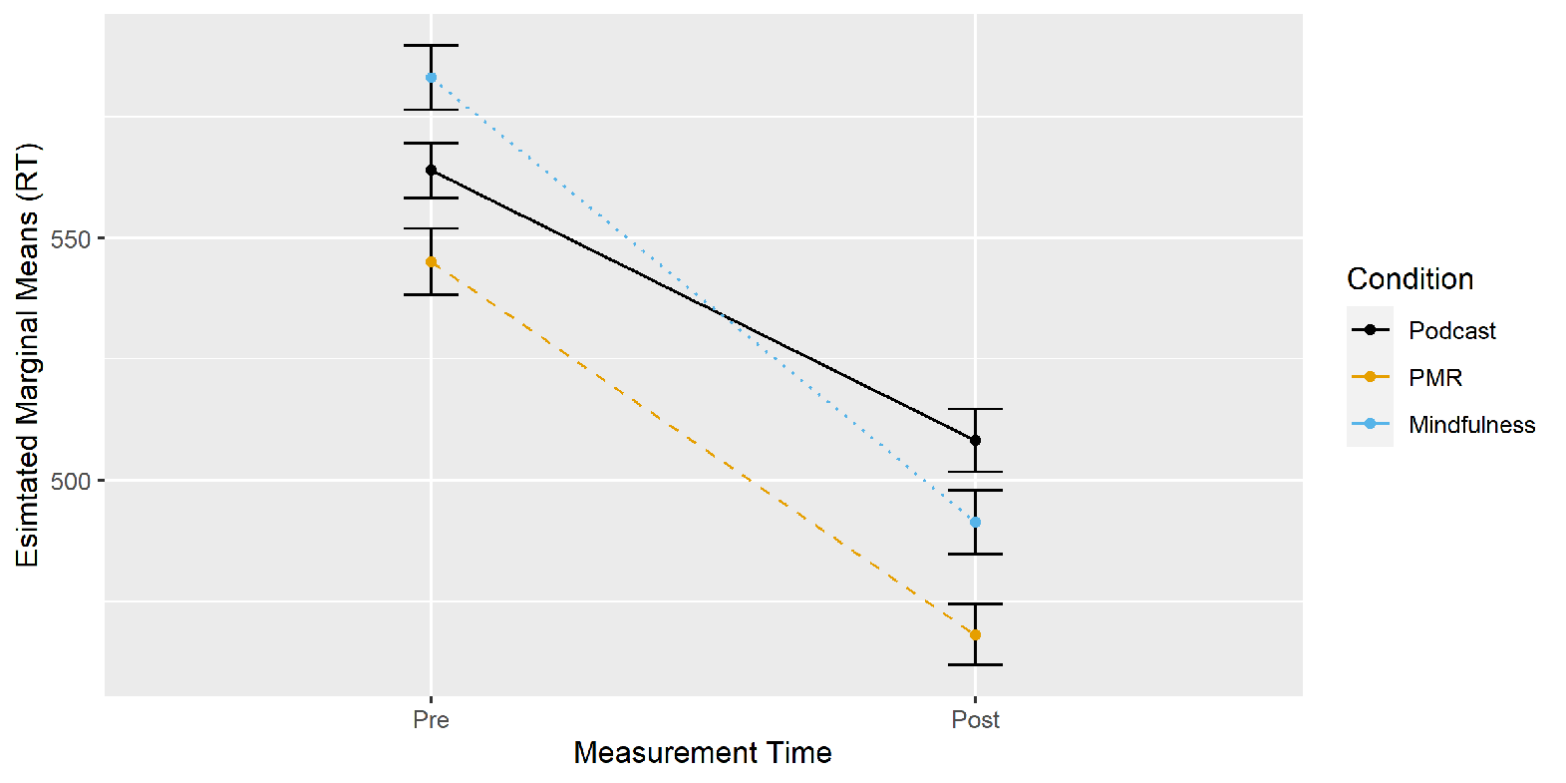
N-Back: Changes in RT from Pre- to Post-Measurement by Condition.

***Figure 4*** displays EMMs for the two-way interaction of time of measurement by condition. Planned comparisons were computed with RT change scores (EMM_t1_ – EMM_t0_) between all conditions (see ***Table 1***). In comparison to the podcast listening group, both mindfulness and PMR exhibited a larger decrease in RT over time of measurement.

Taken together, the results show improved updating for both mindfulness and PMR induction compared to podcast listening and no significant differences between mindfulness and PMR.

#### Accuracy Analysis

Discriminability (*d*’) was analyzed with a model including a fixed effect for age and a three-way interaction between time of measurement, condition and n-back level (see Appendix C, Table C5).

The analysis showed a simple effect of n-back level: 2-back, *β* = –0.48, *SE* = 0.11, *p* < 0.001, and 3-back, *β* = –1.30, *SE* = 0.11, *p* < 0.001, indicating lower *d’* for the 2- and 3-back conditions compared to the 1-back condition. All other simple and main effects were non-significant, *p* ≥ 0.167. All two-way interactions including the factor time of measurement were non-significant, *p* ≥ 0.132, as were all three-way interactions, *p* ≥ 0.155.

Analogous models with errors of omission as the dependent variable (see Appendix C, Table C6) showed a two-way interaction between time of measurement and condition (PMR), *β* = –1.03, *SE* = 0.51, *p* = 0.044. Whereas errors of omission decreased in the podcast group, they increased slightly after PMR; all other interactions including the factor time of measurement were non-significant, *p* ≥ 0.194. EMM contrasts for errors of omission showed no significant differences between groups (see ***Table 1***).

Thus, the results indicate no significant differences between mindfulness and PMR compared to podcast listening regarding discriminability and errors of omission.

### Number-Letter Task

Two participants were removed due to technical difficulties with recording (total data loss: 13.47%; this high level of data loss was partly due to equipment failure. However, the data loss was equally distributed across conditions and across measurement points).

#### Reaction Time

The model included fixed effects for age and accuracy, a random slope for time of measurement by participant and a three-way interaction of time of measurement by condition by switch factor (non-switch as the reference category; see Appendix C, Table C7). The switch factor was included in the three-way analysis to investigate the effect of non-switch versus switch trials and to calculate switch costs for planned comparisons.

##### Simple and Main Effects

The analysis showed a main effect of accuracy, *β* = 26.12, *SE* = 5.54, p < 0.001, with higher RT for correct compared to incorrect trials, and a main effect of age, *β* = 1.42, *SE* = 0.77, *p* < 0.001, with RT increasing as participants’ age increased. Simple effects were present for the switch factor, *β* = 97.76, *SE* = 3.59, *p* < 0.001, with longer RT for switch compared to non-switch trials. There was also a simple effect for time of measurement, *β* = –80.91, *SE* = 4.79, *p* < 0.001, with RT decreasing from pre- to post-measurement.

##### Interactions of Interest

A two-way interaction between time of measurement and switch was found, *β* = –44.77, *SE* = 5.02, *p* < 0.001, indicating a decrease in switch costs from pre- to post-measurement, as well as a significant interaction between time of measurement and condition (mindfulness), *β* = –28.08, *SE* = 6.78, *p* < 0.001, with RT decreasing for mindfulness compared to podcast listening from pre- to post-measurement. The two-way interaction for time of measurement and PMR was non-significant, *p* = 0.092. Additionally, the three-way interaction between time of measurement, condition, and switch was significant for PMR, *β* = 29.40, *SE* = 7.73, *p* < 0.001, indicating that the increase in speed (decrease in RT) for PMR compared to the podcast group was smaller for switch compared to non-switch trials. Thus, the reduction in switch costs from pre- to post-measurement was smaller for PMR compared to the podcast listening group.

Likelihood ratio tests (see Appendix D) showed that the model described above fit significantly better than a model with a two-way interaction of time of measurement by condition only, χ² (5) = 46.01, *p* < 0.001, a model with no interaction terms, χ² (7) = 46.95, *p* < 0.001, and a null model, χ² (13) = 585.47, *p* < 0.001.

**Figure 5:**
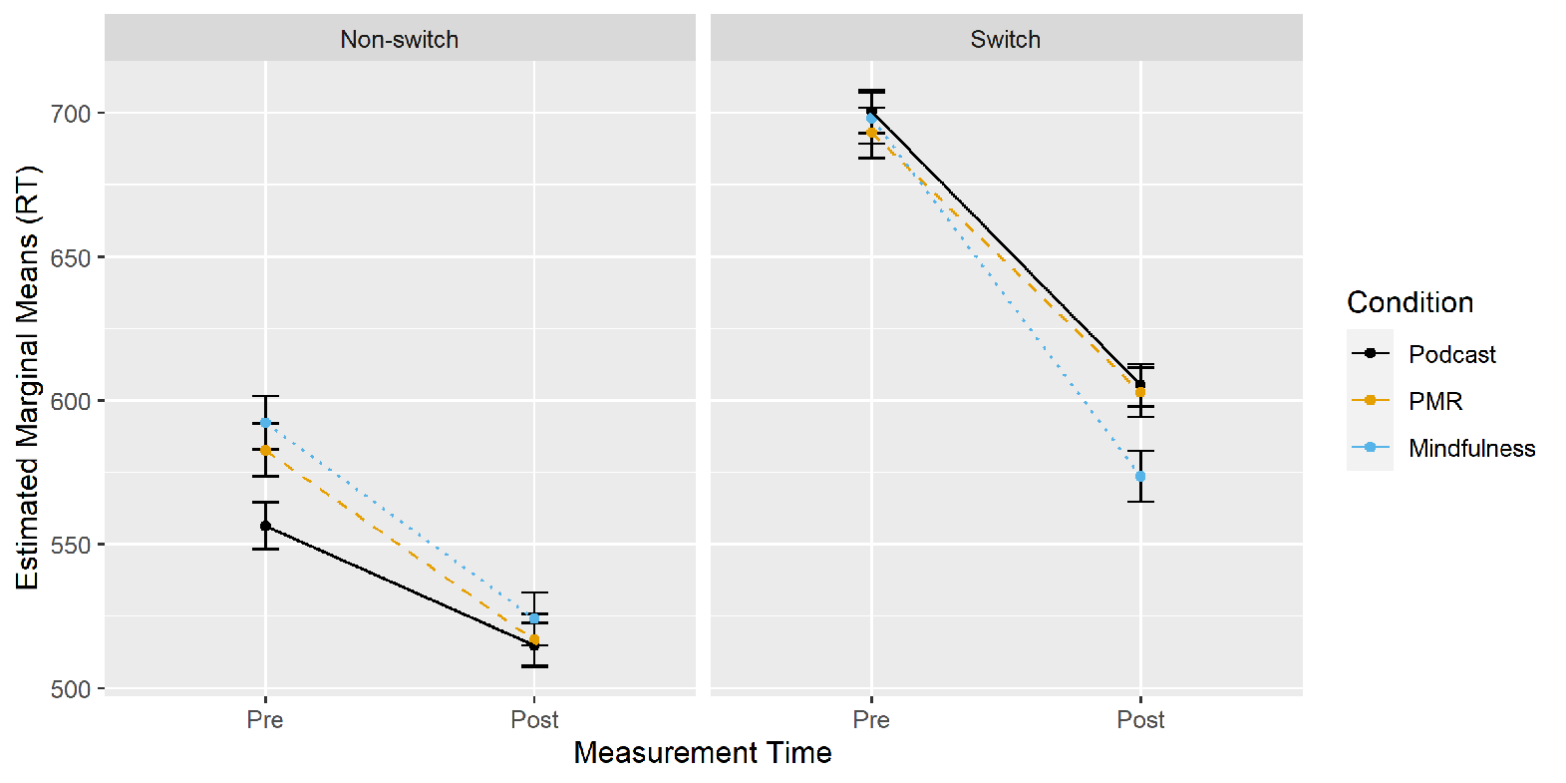
Number-Letter Task: Changes in RT from Pre- to Post-Measurement by Condition and Trial Type.

***Figure 5*** displays EMMs for the three-way interaction between time of measurement, condition and the switch factor. Planned comparisons were calculated for pre-post differences in switch costs (EMM_switch_ – EMM_nonswitch_) between all conditions (***Table 1***). Mindfulness resulted in a larger decrease in switch costs over time than PMR, which resulted in a lesser decrease in switch costs than podcast listening.

Taken together, the results indicate differential effects of mindfulness and PMR compared to podcast listening. The decrease in RT following mindfulness induction was larger than in the podcast group irrespective of the switch factor. However, following PMR, a larger decrease in RT over time compared to podcast listening occurred only for non-switch trials. Analyzing switch costs revealed an improvement in task-switching abilities for mindfulness compared to PMR and a decrease for PMR compared to podcast listening. Switch costs did not differ between mindfulness and podcast listening.

#### Accuracy Analysis

The model included a fixed effect for age, a random slope for time of measurement by participant and a three-way interaction between time of measurement, condition and switch factor, as well as all two-way interactions containing these factors (see Appendix C, Table C8).

There was a significant two-way interaction between time of measurement and switch, *β* = –0.26, *SE* = 0.12, *p* = 0.027, indicating that from pre- to post-measurement, accurate response rates decreased for switch trials compared to non-switch trials. All other two-way interactions including the factor time of measurement were non-significant, *p* ≥ 0.638, as were all three-way interactions, *p* ≥ 0.219.

Taken together, the results indicate no effects on response accuracy from pre- to post measurement for mindfulness or PMR compared to podcast listening.

### Attention Network Task

Two participants were removed from the analysis due to too many missing data points (total data loss after data cleaning: 3%).

#### Reaction Time

Possible effects on attentional networks were tested in separate models including a three-way interaction of time of measurement by condition by target type (executive network) or time of measurement by condition by type of cue (alerting vs. orienting). The network effects were calculated as proposed by Fan et al. (2002).

The model for **the executive network** included fixed effects for cue (with no cue as the reference category), age and accuracy; a random slope for time of measurement by participant and a three-way interaction of time of measurement by condition by target type (congruent vs. incongruent, with congruent trials as the reference category; see Appendix C, Table C9). Target type was included in the three-way interaction to investigate the effect of congruent versus incongruent trials and to calculate the executive network score for planned comparisons.

##### Simple and Main Effects

There was a main effect of age, *β* = 3.39, *SE* = 0.53, *p* < 0.001, with RT increasing with participants’ age, and a main effect of accuracy, *β* = 82.79, *SE* = 1.32, *p* < 0.001, with higher RT in accurate compared to inaccurate trials. Simple effects were present for target type, *β* = 103.58, *SE* = 0.90, *p* < 0.001, reflecting slower RT for incongruent compared to congruent trials, and time of measurement, *β* = –37.82, *SE* = 2.55, *p* < 0.001, reflecting a decrease in RT from pre- to post-measurement.

##### Interactions of Interest

A two-way interaction was found between time of measurement and target, *β* = –21.44, *SE* = 1.66, *p* < 0.001, indicating that RT decreased to a larger degree from pre- to post-measurement in incongruent trials than in congruent trials. All other two-way interactions including the factor time of measurement were non-significant, *p* ≥ 0.075. Furthermore, the model yielded a significant three-way interaction between time of measurement, condition, and target type for both mindfulness, *β* = –12.66, *SE* = 2.58, *p* < 0.001, and PMR, *β* = –14.02, *SE* = 2.57, *p* < 0.001. For both mindfulness and PMR, RTs for incongruent trials improved to a larger degree from pre- to post-measurement compared to congruent trials and compared to the podcast listening condition, indicating improved conflict resolution after both inductions.

Likelihood ratio tests (see Appendix D) showed that the model described above fit significantly better than a model with a two-way interaction of time of measurement by condition only, χ² (5) = 211.71, *p* < 0.001, a model with no interaction terms, χ² (7) = 212.05, *p* < 0.001, and a null model, χ² (16) = 11825, *p* < 0.001.

**Figure 6:**
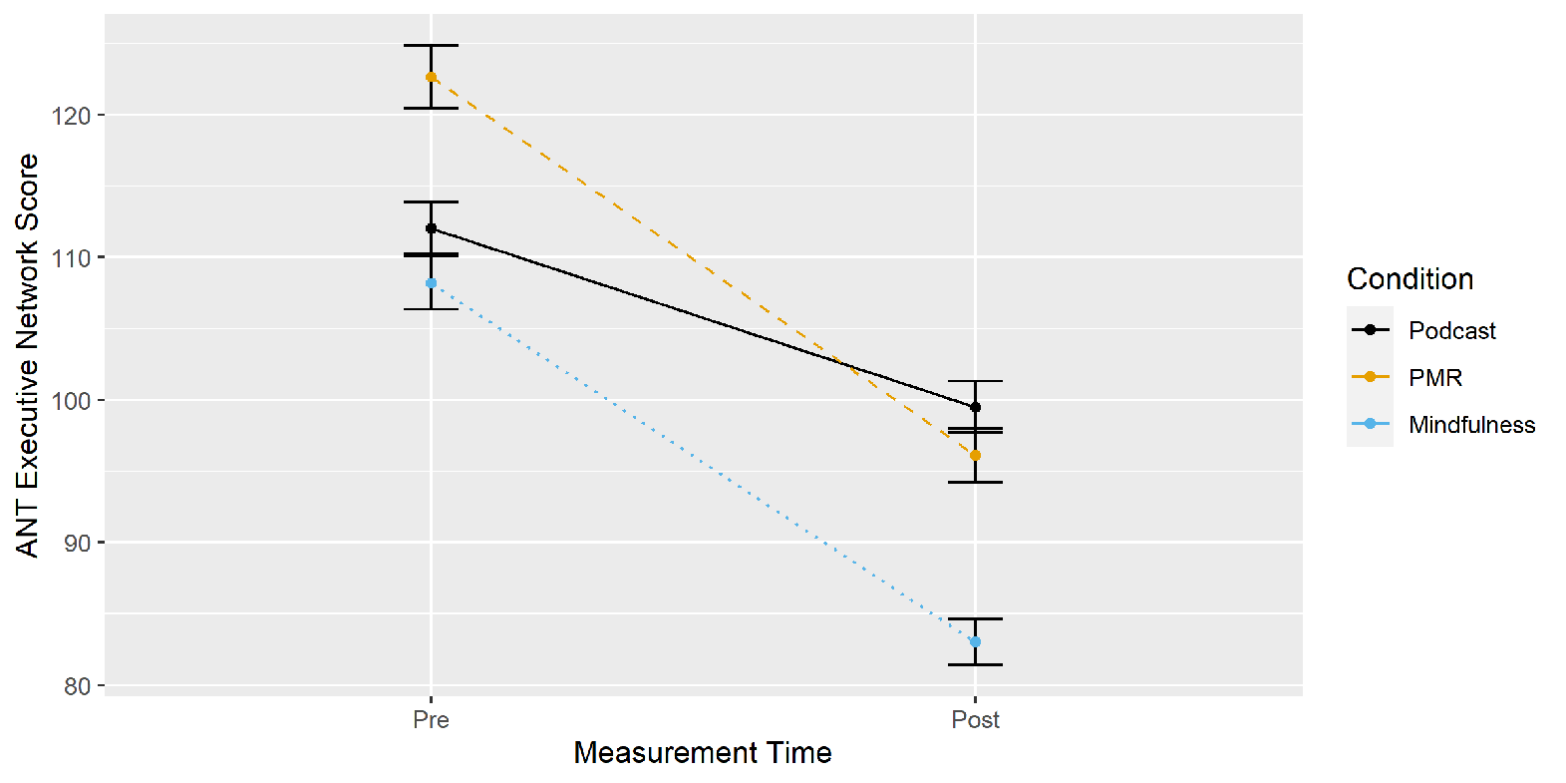
ANT: Executive Network Score from Pre- to Post-Measurement by Condition.

***Figure 6*** displays conflict effects for the three-way interaction of time of measurement, condition and target. Executive network scores (EMM_incongruent_ – EMM_congruent_) are displayed on the y-axis. Planned comparisons of pre-post differences (EMM_t1_ – EMM_t0_) between conditions (***Table 1***) showed that both mindfulness and PMR resulted in an improvement compared to podcast listening.

The results suggest that RTs for incongruent compared to congruent trials improved to a larger degree following both mindfulness and PMR compared to podcast listening. Accordingly, both inductions improved conflict resolution compared to the podcast group. Mindfulness did not differ significantly from PMR.

The model for **the alerting and orienting networks** included fixed effects for target, age and accuracy, a random slope for time of measurement by participant and a three-way interaction of time of measurement by condition by cue (see Appendix C, Table C10). Cue type was included in the three-way interaction to investigate the differential effects of the cue versus no-cue trials and to calculate the alerting and orienting network scores for planned comparisons.

##### Simple and Main Effects

There was a main effect of age, *β* = 3.41, *SE* = 0.55, *p* < 0.001, with RT increasing with participants’ age, and a main effect of accuracy, *β* = 84.13, *SE* = 1.34, *p* < 0.001, with higher RT in accurate compared to inaccurate trials. Additionally, simple effects were found for cue types: middle cue, *β* = –11.39, *SE* = 1.17, *p* < 0.001, spatial cue, *β* = –12.67, *SE* = 1.16, *p* < 0.001, double cue, *β* = –11.85, *SE* = 1.18, *p* < 0.001. Compared to no cue trials, RT improved in trials in which the aforementioned cues were presented. A simple effect was found for time of measurement, *β* = –34.76, *SE* = 3.80, *p* < 0.001, reflecting a decrease in RT from pre- to post-measurement.

##### Interactions of Interest

All two-way interactions including time of measurement were non-significant, *p* ≥ 0.160. Additionally, the three-way interaction between time of measurement, condition, and cue was significant for mindfulness and middle cue, *β* = 12.36, *SE* = 3.49, *p* < 0.001, and spatial cue, *β* = 9.23, *SE* = 3.49, *p* = 0.008, indicating that for both cues, compared to no cue trials, a larger decrease in RT from pre- to post-measurement was found for podcast listening compared to mindfulness. For PMR, a three-way interaction was found with spatial cue, *β* = 12.49, *SE* = 5.09, *p* = 0.014, and double cue, *β* = 9.54, *SE* = 4.39, *p* = 0.030, indicating a larger decrease in RT from pre- to post-measurement compared to no cue trials for the podcast group compared to PMR; *p* ≥ 0.110.

Likelihood ratio tests (see Appendix D) showed that the model described above fit significantly better than a model with a two-way interaction of time of measurement by condition only, χ² (15) = 27.42, *p* = 0.026, a model with no interaction terms, χ² (17) = 27.75, *p* = 0.048, and a null model, χ² (26) = 11641, *p* < 0.001.

**Figure 7:**
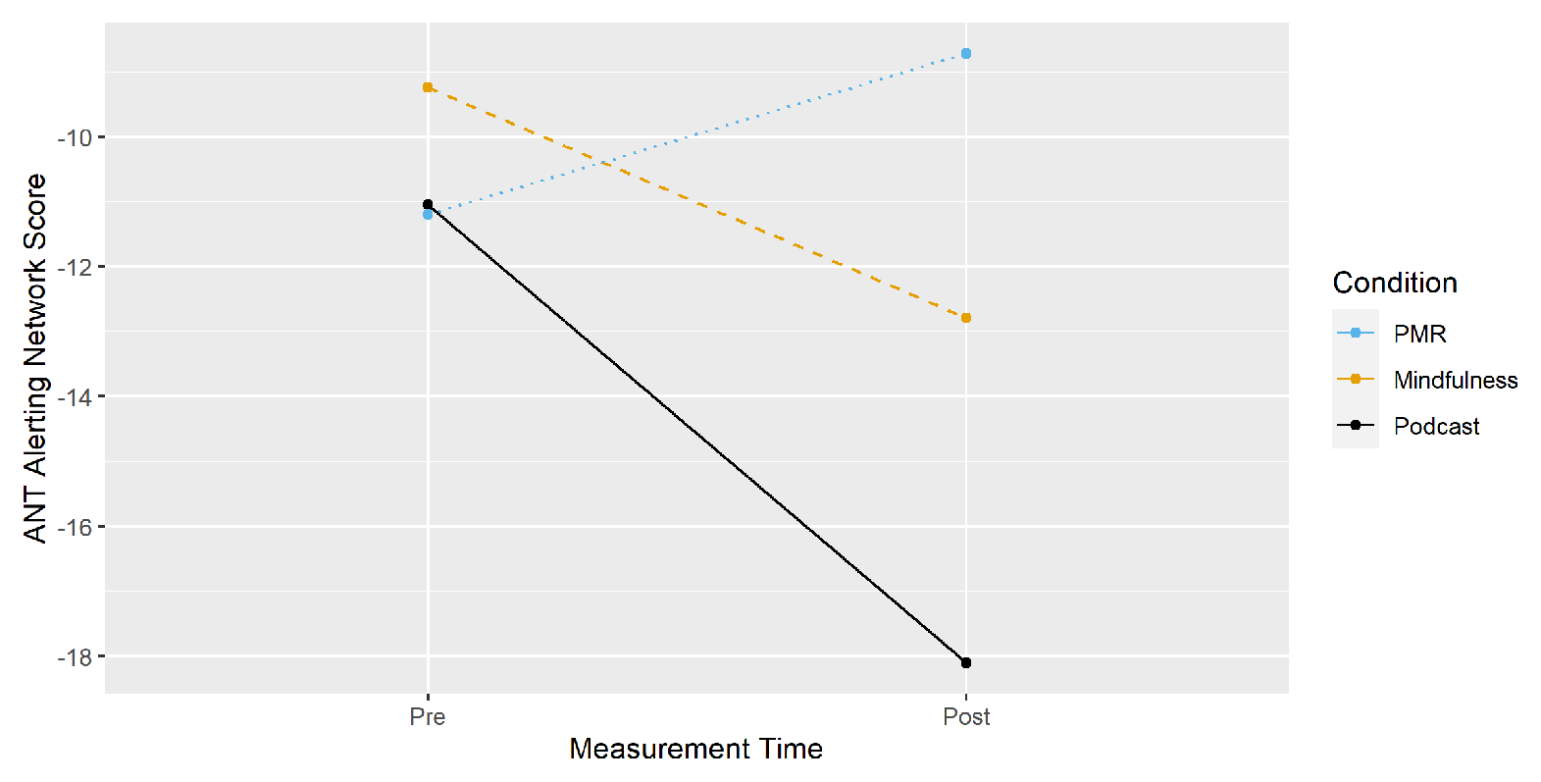
ANT: Alerting Network Score from Pre- to Post-Measurement by Condition.

**Figure 8:**
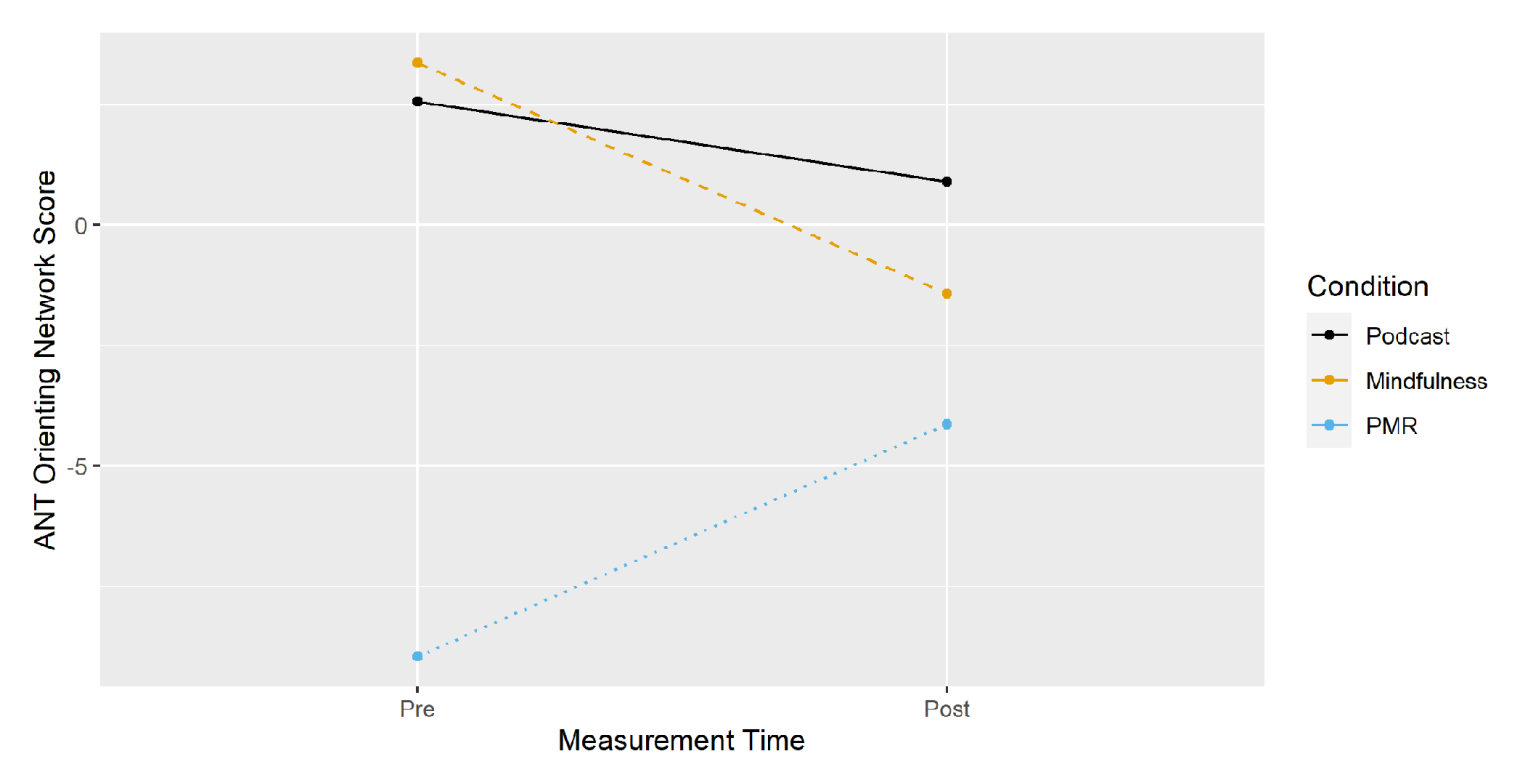
ANT: Orienting Network Score from Pre- to Post-Measurement by Condition.

Alerting and orienting effects for the three-way interaction of time of measurement, condition and cue (Alerting Network Score = EMM_doublecue_ – EMM_nocue_; Orienting Network Score = EMM_spatialcue_ – EMM_centercue_) are displayed in ***Figure 7*** and ***Figure 8***, with alerting and orienting network scores on the y-axes respectively. Planned comparisons of pre-post differences (EMM_t1_ – EMM_t0_) between conditions (***Table 1***) showed no significant effects.

The results suggest a general advantage of cue over non-cue conditions and a benefit for the podcast compared to both induction conditions in responding to individual cue conditions. However, planned comparisons for the calculated alerting and orienting network scores did not reveal any differential effects between groups.

#### Accuracy Analysis

Possible effects were tested in separate models including a three-way interaction of time of measurement by condition by target (executive network) or time of measurement by condition by cue (alerting and orienting).

The model for **the executive network** included fixed effects for age and cue type, a random slope for time of measurement by participant and a three-way interaction between time of measurement, condition and target type, as well as all two-way interactions containing these factors (see Appendix C, Table C11).

There was a significant two-way interaction for target type by time of measurement, *β* = 0.27, *SE* = 0.13, *p* = 0.034, indicating that participants’ accuracy in incongruent trials compared to congruent trials improved from pre- to post-measurement. All other two-way interactions including the factor time of measurement were non-significant, *p* ≥ 0.141, as were all three-way interactions, *p* ≥ 0.693.

Taken together, the results for conflict resolution indicate no influence of either mindfulness or PMR induction compared to podcast listening on response accuracy within the executive network.

The model **for the alerting and orienting networks** included fixed effects for age and target type, a random slope for time of measurement by participant and a three-way interaction between time of measurement, condition and cue type, as well as all two-way interactions containing these factors (see Appendix C, Table C12).

All two-way interactions including the factor time of measurement were non-significant, *p* ≥ 0.118, as were all three-way interactions, *p* ≥ 0.167.

Taken together, there was no indication of any effects of mindfulness or PMR induction compared to podcast listening on response accuracy within the alerting or orienting networks.

### Questionnaires

Two-way repeated-measures analyses of variance (ANOVA) were carried out on the PANAS and MAAS scores. Results showed no significant main effects or interactions for negative affect (PANAS) or MAAS scores (p ≥ 0.297). Main effects for positive affect were also non-significant, however, there was a significant interaction for group and time of measurement. Therefore, GLMMs were run to test for possible influences of positive affect on reaction time on all cognitive tasks utilized. No significant results were found (p ≥ 0.096). Further information about the scales and statistical results are described in Appendix A.

## Discussion

While scientific models of mindfulness meditation identify improved attention and executive functioning as possible mechanisms, there is ongoing discussion about how much practice is required to spark mindfulness-specific effects (Fell et al., 2010). In particular, it is unclear whether the effects of short mindfulness inductions are specific to mindfulness or can also be achieved through other means such as relaxation. We addressed this research question by employing a randomized controlled pre-post design, contrasting mindfulness with a relaxation induction as an active control condition and listening to podcasts as a passive control condition. Our results revealed differential effects of mindfulness compared to PMR and podcast listening on the executive functions of set maintenance/inhibition and set shifting/switching. However, specific *benefits* of mindfulness arose only for the switching task, and both inductions yielded comparable benefits regarding updating/working memory and attention networks. We discuss our findings for each assessed executive or attentional function below. We relate our findings to studies that employed similar assessment tasks but longer periods of practice and to mechanisms proposed by models of mindfulness discussed in the introduction.

The mindfulness induction improved **inhibition** latencies from pre- to post-measurement compared to both PMR and podcast listening. Interestingly, inducing relaxation prolonged latencies in the shortest ISI but improved them in longer ISIs. Taking response quality into account showed that PMR induction improved discriminability compared to podcast listening and reduced errors of omission compared to both mindfulness and podcast listening. These dissimilar effects suggest differential mechanisms underlying mindfulness and relaxation, and some advantages of mindfulness – albeit not exclusively. Relatedly, Wenk-Sormaz (2005) found improved inhibition as reflected in reduced Stroop interference in RTs after 20 minutes of focused attention meditation in comparison to cognitive control tasks and a passive rest condition. In a correlational study, Schmertz et al. (2009) found an association between higher scores on trait mindfulness (MAAS) and fewer omissions in a CPT-II. Therefore, whereas the benefits of mindfulness induction for inhibition latency emerge instantly (but not after relaxation induction), effects on response quality did not and may require longer practice or high trait mindfulness.

Mindfulness induction and PMR both resulted in improved **updating** latencies compared to podcast listening but no effects on updating accuracy. Similarly, Johnson et al. (2015) found no differences in response speed or extended hit rate in a modified 2-back task after a short meditation, a sham meditation or listening to an audiobook. Zeidan et al. (2010) likewise found no benefits of a brief mindfulness intervention over four training sessions compared to audiobook listening in accuracy in a modified 2-back task, but only regarding extended hit runs (i.e. number of correct responses in a row).^4^ However, when examining the effects of longer mindfulness interventions on working memory, Basso et al. (2019) reported that eight weeks of meditation improved response accuracy on the n-back task compared to a podcast listening condition. Therefore, we conclude that specific effects of mindfulness on updating and working memory capacity like those proposed by Jha et al. (2019) may only unfold over time and would need consolidation through practice.

Analyzing switch costs revealed an improvement in **task switching** for mindfulness compared to PMR and a decline in task switching for PMR compared to podcast listening. In line with our findings of improved overall speed after mindfulness, Jankowski and Holas (2020) found improved response speed across switch and non-switch trials after mindfulness induction compared to a worry induction and free mind-wandering in a study investigating the effects of induced negative affect. However, these authors reported no reduction in switch costs for mindfulness compared to the control conditions. Chambers et al. (2008) investigated the effects of a 10-day mindfulness retreat on the internal switching task, finding an improvement in RTs for the mindfulness condition compared to a wait-list control condition. Employing the number-letter task in a study on the effects of a mindfulness intervention over seven bi-weekly sessions, Wimmer et al. (2020) found that mindfulness but not the active control condition (awareness training) resulted in a greater overall speed improvement than the passive control condition (no training). Based on our findings and previous studies, we conclude that the benefits of mindfulness for switch costs rely on specific mechanisms that can be differentiated from the effects of relaxation even after brief inductions of a mindful state.

Our results show that both mindfulness and PMR similarly improved conflict resolution through the **executive network** and thus yielded no evidence for specific effects of a brief mindfulness induction. However, Tang et al. (2007), investigating a five-day integrative body-mind training compared to PMR, and Kwak et al. (2020), investigating an intensive four-day mindfulness retreat compared to non-guided relaxation, found effects on conflict resolution after meditation but not after relaxation practice. Specific benefits of mindfulness may therefore require more practice time to unfold. Neither mindfulness nor PMR affected the performance of the **alerting and orienting networks** in the present study. Examining effects of longer mindfulness interventions on attentional networks, Jha et al. (2007) showed an improvement in orientation latencies after eight weeks of MBSR training compared to a passive control condition. Again, these effects may require more practice time to unfold.

It appears worth noting that for all functions assessed in the present study (except alerting and orienting, which were not affected by either induction), we found an improvement in reaction times following mindfulness induction compared to the podcast listening group. While participants increased the speed of their responses from pre- to post measurement, they maintained their rates of correct answers. That is, the mindfulness induction did improve performance. However, this improvement in reaction times was also found following PMR, except for the short ISI inhibition and task switching, indicating that a short relaxation induction also appears to enhance and/or reduce interference with the respective processes. Thus, specific benefits of mindfulness compared to relaxation apart from task switching seem to require longer periods of practice.

Relating our results and those of studies with longer periods of practice to mechanisms proposed by models of mindfulness shows that these models correctly predict several outcomes, such as better inhibition and task switching (Bishop et al., 2004), better executive attention (Malinowski, 2013), or improvement in working memory (Jha et al., 2019) following mindfulness practice. However, not all predicted benefits of mindfulness practice turned out to be specific. In light of our findings of a mindfulness-specific benefit for task switching as well as partly specific improvement in inhibition, executive functions may indeed lie at the core of effects that can be considered specific to mindfulness practice and are clearly distinguishable from effects of relaxation even after short inductions. However, we found comparable effects for mindfulness and relaxation for the executive attention network, which is proposed to be involved in disengagement from mind wandering during practice. Also, we found that both mindfulness and relaxation improved working memory capacity. Furthermore, no effects for alerting or orienting arose. Therefore, this discussion shows that the dose-response relation and the specificity of mindfulness-based mechanisms need to be further specified in theoretical approaches. The phenomenological matrix approach by Lutz et al. (2015) might be considered a step in this direction. In order to systematize the phenomenological experience of mental states in general, that is, inside as well as outside meditation practice, Lutz et al. (2015) proposed three dimensions along which states of mind can be arranged. These are object orientation (which is high in FAM but also in states of craving), dereification (i.e., the degree to which one considers states of mind as passing mental processes rather than valid representations of reality; dereification is low in states of craving and high in intensely practiced OMM), and meta-awareness, comprising awareness of the task set and at the same time of the larger context of one’s subjective experience. Mental states on these dimensions can further be qualified in terms of how open vs. focused they are, how clear and stable, and how much effort goes into maintaining them (the latter of which is, for example, low in craving and high in a novice’s focused attention meditation). This categorization also makes it possible to clearly distinguish qualities of the mental states of novice and expert practitioners. For example, novice practitioners will need to put in more effort to maintain their meditative state and not get carried away through mind wandering than experts, and when practicing focused attention meditation (FAM), they may only achieve a high level of object orientation after a certain period of practice (Lutz et al., 2008; Lutz et al., 2015). Object orientation during PMR, on the other hand, may be easier to achieve without much practice, as attentional focus is grounded in immediate proprioceptive feedback through the alternating constriction and relaxation of muscles. However, both meditation with a focus on the breath (e.g. FAM) and PMR require the practitioner to both focus their attention to somatosensory experiences and narrow their attentional scope to the object that is to be observed (i.e. the quality of narrow aperture, according to Lutz et al., 2015). Selecting active control groups along these dimensions and also controlling for changes in dimensions over practice time may help us to better understand which mindfulness effects are specific to how much practice.

## Conclusion

Our results suggest partly differential, partly overlapping mechanisms of mindfulness compared to relaxation induction on attention and executive functions and are therefore partly in line with Fell et al.’s (2010) proposal that the cognitive benefits found for initial steps of meditative development may be not specific to the practice. However, based on our results and previous studies, we propose that differential effects of mindfulness across attentional processes and executive functions may become apparent after longer practice.

### Limitations and Future Research

We chose to deliver the mindfulness and PMR instructions in person to support participants’ engagement with the practice. However, in-person instructions are less standardized than pre-recorded instructions, meaning that our choice may have reduced internal validity (for a related discussion, see Cavanagh et al., 2014; Fish et al., 2016).

In our design, we opted to screen for participants who had not engaged in mindfulness practice within the last three months. While the exact dose-response relationship as well as the longevity of effects following mindfulness trainings are still up for debate, there is consensus that continued practice is necessary to maintain these effects (e.g. Fell et al., 2010; Malinowski, 2013). Nevertheless, future studies may wish to consider longer periods of non-practice or test only naive participants to exclude the possibility of reactivating effects of previous meditation practice.

Based on our results and previous studies, we argue that benefits specific to mindfulness practice may require training to consolidate. This raises the question of the dosage-response relationship between mindfulness, attention, and executive functioning. Therefore, it would be interesting to investigate such changes by increasing the training dosage in small increments and employing repeated testing.

We controlled for possible influences of trait mindfulness and emotions, but more detailed information about how calm, relaxed and/or mindful participants actually felt after the inductions might be interesting to assess as a manipulation check in future studies, e.g. using the Smith Relaxation States Inventory 3 (SRSI3; Smith, 2017 & 2020; German version: Vieth et al., 2020).

Replicating our results with further measures of attention and executive control would obviously be desirable. Furthermore, active control conditions are essential for studying the mechanisms underlying mindfulness induction, as advantages compared to passive conditions alone leave open what exactly the transient starting points of longer-lasting practice effects are.

## Additonal Files

The additonal files for this article can be found as follows:

10.5334/joc.205.s1 Appendix A. Supplementary Material on Questionnaires Used to Control for Possible Influences of Mood on Attentional and Executive Control.

10.5334/joc.205.s2 Appendix B. Supplementary Material Containing Instructions for the Mindfulness Meditation and Progressive Muscle Relaxation.

10.5334/joc.205.s3 Appendix C. Supplementary Material Containing Statistical Analysis and Results Tables.

10.5334/joc.205.s4 Appendix D. Generalized Linear Mixed Model Comparison of Measures of Attentional Control and Executive Functioning.

## Footnotes

While this variant of the CPT may also assess aspects of vigilance, it mainly requires inhibition. Throughout the task, the participant is instructed to produce a readiness to withhold a response once a rare target (X) is detected. This differs from classical CPTs in which the participant is instructed to execute a response when a frequent target (X) is detected (for a discussion see Ballard, 2001).

Re-running our analysis without ISI did not change the significance of any interactions of interest and did not provide a better fit to the data.

Re-running our analysis without n-back level did not change the significance of any interactions of interest and did not provide a better fit to the data.

Supplementary analyses of extended hit runs in the n-back task revealed that PMR exceeded both the mindfulness and passive control conditions in hit run gains from pre- to post-measurement. Such a gain was also present for mindfulness compared to the passive control group, but did not reach significance.
