# Supplementary material for: Mechanisms Underlying Cognitive Effects of Inducing a Mindful State
Source: J Cogn. 2022 Jan 21;5(1):13. doi: 10.5334/joc.205 (PMC9400668; doi:10.5334/joc.205)
Supplement: Appendix A. — Supplementary Material on Questionnaires Used to Control for Possible Influences of Mood on Attentional and Executive Control. [file joc-5-1-205-s1.pdf]

Appendix A

**Supplementary Material on Questionnaires Used to Control for Possible Influences of Mood on Attentional and Executive Control**

The PANAS is a questionnaire measuring positive and negative emotions on two respective subscales and consists of 20 items in total, ten describing positive and ten describing negative affective states. Participants are asked to indicate on a 5-point Likert-type scale how well a certain affective state (e.g. “scared”) applies to them currently. The Mindful Attention and Awareness Scale is a 15-item scale used to measure awareness of and attention to the present moment.

In order to control for possible effects of affect, two-way repeated-measures analyses of variance (ANOVA) were carried out on the PANAS scores. The results showed ~~changes in mood~~no significant main effect of condition, $F(2, 76) = 0.385$, $p = 0.819$, or time, $F(2, 76) = 1.240$, $p = 0.297$. Negative affect did not change significantly over time between groups~~ related to ~~, $F(2, 76) = 0.580$, $p = 0.563$, but significant changes in positive affect over time were present between groups, $F(2, 76) = 4.481$, $p = 0.015$~~, but not for negative affect F(2, 76) = 0.579, p = 0.563.~~ . Separate GLMMs for each cognitive task with RT as the dependent variable and positive affect scores per measurement point as independent variables suggested no significant influence of positive affect on reaction time for all tasks described in the results section~~.~~, all p’s > 0.096. Similarly, to control for possible differences in trait mindfulness between pre- and post-measurement as well as between groups, a two-way repeated measures ANOVA was conducted, revealing no main effect of time, $F(2, 78) = 0.867$, $p = 0.355$, as well as no pre-post differences in MAAS scores between groups, $F(2, 78) = 1.100$, $p = 0.338$.
