# Supplementary material for: Mechanisms Underlying Cognitive Effects of Inducing a Mindful State
Source: J Cogn. 2022 Jan 21;5(1):13. doi: 10.5334/joc.205 (PMC9400668; doi:10.5334/joc.205)
Supplement: Appendix B. — Supplementary Material Containing Instructions for the Mindfulness Meditation and Progressive Muscle Relaxation. [file joc-5-1-205-s2.pdf]

Appendix B

Supplementary Material Containing Instructions for the Mindfulness Meditation and Progressive Muscle Relaxation

Inductions were read to the participants in German. Below are translated versions of the instructions.

Mindfulness Meditation

The mindfulness instructions focused on being present in and aware of the present moment, observing one's flow of breath without interfering with it, observing and letting go of thoughts and emotions that arise, and overall acceptance of the present moment.

Breathing Meditation Instructions

Sit down in a way that allows you to sit for a while in a well-balanced position. Straighten up slightly. Some people imagine a golden thread at the crown of their head that is pulling their head up.

(If you like,) close your eyes. If you tend to get sleepy, fixate on any point in the room in front of you.

Feel where your body is in contact with the chair, where it is supported and sustained.

1-minute pause

Now focus your attention on your breath. Breathe in and breathe out and be aware of this process of breathing. Become aware of your breath, where your body moves in the rhythm of your breath, and how. In the chest, in the stomach, or some other place?

2-minute pause

Feel the ever-changing sensations as the current of your breath flows in and out of the body.

3-minute pause

Allow your body to breathe with its own rhythm. You don't need to change or monitor anything. You are an observer of the processes that come naturally, from one moment to the next.

1-minute pause

Thoughts of all kinds may come to your mind. This is totally fine. Once you've taken note of this, simply direct your attention back to your breathing.

1-minute pause

If you are being critical of yourself, notice this and bring your attention back to your breathing. Be patient with yourself.

2-minute pause

Now take in the complete breathing process once again:

On the inhale, how breath flows in through the nose and lifts the chest and stomach.

On the exhale, how it flows out from the nose and then lowers the chest and belly and the pause after the exhale.

3-minute pause

Broaden your attention again to your whole body and to the space where you are sitting. Let your breath flow again without observing it.

2-minute pause

When you feel ready, open your eyes or let your gaze wander around the room. Be ready and awake for what is next in store for you.

Progressive Muscle Relaxation

During PMR, the participant is instructed to contract a specific muscle group (e.g. the upper thighs) for five to ten seconds during inhalation, and to let go of the tension during exhalation. The instructions start with rounds of contraction and release focused on the lower extremities and gradually progress upwards through the body. Between muscle groups, subjects are asked to take ten to 20 seconds for relaxation and to focus on changes in their physiological experience when releasing the tension.

Progressive Muscle Relaxation Instructions

Notes

Tension: 5 seconds.

Pause between steps: 10 seconds.

Introduction

Tighten each muscle of your body one by one for about 5 seconds, not too much but just until you feel a slight stretch, and hold the tension. Then release the tension without moving around much. Next, consciously pay attention to the feeling of relaxation for about 10 seconds.

If you don't feel the relaxation the first time, repeat it again. While tensing each muscle, try to keep all other muscles as relaxed as possible. Concentrate solely on the particular muscle group you are tensing.

Posture

Sit up straight. Your head is straight between your shoulders and your legs are together. Your arms are resting on your thighs.

Your feet are firmly on the floor.

Unless you find it uncomfortable, close your eyes. If you become slightly drowsy, fixate on a point about 2m in front of you on the floor.

Now take a few deep breaths and let your body become loose and pleasantly heavy. Take your time here.

1. Now clench your right hand into a fist and tighten it. Count slowly from 1 to 5... and release the tension. Now enjoy the feeling of relaxation.
2. Now clench your left hand into a fist, count slowly from 1 to 5....and then relax again.
3. Now tense your forearm muscles by reaching up your hands. Your forearms should remain on your thighs. Hold the tension... and relax again. Feel the relaxation.
4. Now tense your forearm muscles by bending your elbows with open hands. Hold the tension... and relax again.
5. Now crunch up your forehead. While doing so, open your eyes wide. Raise your eyebrows so that your forehead becomes wrinkled, hold the tension... and relax again. If you like, close your eyes again. Continue breathing calmly and relaxed. Enjoy the feeling of relaxation.
6. Now draw your eyebrows together so that a vertical frown line appears on your forehead. Hold the tension...and relax again.
7. Now close your eyes more firmly and count slowly from 1 to 5.... hold the tension...and relax again. Continue to breathe in a relaxed manner.
8. Now press your lips together without clenching your teeth. Hold the tension...and relax again.
9. Now press your tongue against the roof of your mouth. Hold the tension...and relax again. Let your tongue lie loosely in your mouth. Your breath is flowing calmly and relaxed.
10. Now clench your teeth, hold the tension... and relax again. Enjoy the feeling of relaxation.
11. Now tilt your head down to your right shoulder. Hold the tension...and relax again.
12. Now tilt your head down to your left shoulder. Hold the tension... and relax again. Breathe calmly and relaxed.
13. Now pull your shoulders up to your ears. Hold the tension...and relax again.
14. Now press your shoulder blades together towards the back of your spine. Hold the tension... and relax again.
15. Now inhale deeply so that your chest expands. Hold your chest like this and continue breathing lightly. Hold the tension. Then let your chest collapse .... and relax again. Breathe calmly and relaxed once again.
16. Now push your stomach out and hold it for a moment while continuing to breathe. Hold the tension. Then pull your stomach in... And relax again.

17. Now tense the muscles in your buttocks. Hold the tension... and relax again.

18. Now tense your thighs by pressing your heels into the floor and lifting your toes off the floor. Hold the tension...and relax again.

19. Tense your calves by pressing your feet down onto the floor. Not too hard. Hold the tension... and relax again.

20. Inhale again in a completely relaxed way... and exhale... Repeat this five times... When you feel ready, slowly open your eyes.
