# Supplementary material for: Mechanisms Underlying Cognitive Effects of Inducing a Mindful State
Source: J Cogn. 2022 Jan 21;5(1):13. doi: 10.5334/joc.205 (PMC9400668; doi:10.5334/joc.205)
Supplement: Appendix C. — Supplementary Material Containing Statistical Analysis and Results Tables. [file joc-5-1-205-s3.pdf]

Appendix C

Supplementary Material Containing Statistical Analysis and Results Tables

Statistical Analyses

Analyses were carried out using the R statistics software (R Core Team, 2020). Generalized linear models were fitted using the package ‘lme4’ (Bates, Maechler, Bolker & Walker, 2015) and p-values were obtained with the package ‘lmerTest’ (Kuznetsova, Brockhoff & Christensen, 2017). Estimated marginal means were calculated using the ‘emmeans’ package by Lenth (2020). All plots were created with the ‘ggplot2’ package by Wickham (2016).

Contrast Coding

For all models, the measurement time factor was coded as 0 = pre-measurement and 1 = post-measurement. For the experimental condition factor, the non-treatment control condition was coded as the reference category (treatment coding). The effect of age was controlled by adding the participant’s age (grand-mean centered) as a fixed effect. For accuracy, non-accurate trials were coded as the reference category.

Table C1

Generalized Linear Mixed Model of RT During the Continuous Performance Task

Predictors	*Estimates*	*SE*	*95% CI*	*p*
(Intercept)	259.74	2.76	254.33 – 265.16	**<0.001**
Time	-11.74	2.91	-17.44 – -6.05	**<0.001**
$\text{Condition}_{\text{Mindfulness}}$	-7.44	3.34	-13.98 – -0.90	**0.026**
$\text{Condition}_{\text{PMR}}$	2.80	4.58	-6.17 – 11.76	0.541
ISI_{2000}	15.26	0.80	13.70 – 16.83	**<0.001**
ISI_{4000}	49.13	0.88	47.41 – 50.86	**<0.001**
$\text{Age}_{\text{Grand Mean Centered}}$	2.13	0.42	1.31 – 2.95	**<0.001**
Time : $\text{Condition}_{\text{Mindfulness}}$	-15.04	2.80	-20.54 – -9.54	**<0.001**
Time : $\text{Condition}_{\text{PMR}}$	-0.05	2.93	-5.80 – 5.70	0.986
Time : ISI_{2000}	6.98	1.47	4.11 – 9.85	**<0.001**
Time : ISI_{4000}	1.04	1.64	-2.18 – 4.26	0.527
$\text{Condition}_{\text{Mindfulness}}$: ISI_{2000}	-7.27	1.53	-10.25 – -4.28	**<0.001**
$\text{Condition}_{\text{Mindfulness}}$: ISI_{4000}	-12.00	1.81	-15.55 – -8.46	**<0.001**
$\text{Condition}_{\text{PMR}}$: ISI_{2000}	-4.69	1.59	-7.80 – -1.57	**0.003**
$\text{Condition}_{\text{PMR}}$: ISI_{4000}	-4.81	1.92	-8.58 – -1.04	**0.012**
Time : $\text{Condition}_{\text{Mindfulness}}$: ISI_{2000}	8.04	2.23	3.67 – 12.42	**<0.001**
Time : $\text{Condition}_{\text{Mindfulness}}$: ISI_{4000}	4.51	2.79	-0.96 – 9.97	0.106
Time : $\text{Condition}_{\text{PMR}}$: ISI_{2000}	-17.11	2.57	-22.14 – -12.08	**<0.001**
Time : $\text{Condition}_{\text{PMR}}$: ISI_{4000}	-21.63	2.83	-27.17 – -16.09	**<0.001**

Random Effects		*Model Specifications*	
τ_{00} Subject	155.50	Observations	46731
τ_{11} Subject : Time	238.69	Df	46708
ρ_{01} Subject	-0.06	Deviance	536141.3
N Subject	78	AIC	536187.3
		log-Likelihood	-268070.6

Note. P-values for fixed effects were calculated using Satterthwaites approximations. Confidence Intervals have been calculated using the Wald method. Model fit by maximum likelihood (Laplace Approximation); family: inverse.gaussian (identity).

Table C2

Linear Regression of d' During the Continuous Performance Task

Predictors	*Estimates*	*SE*	*95% CI*	*p*
(Intercept)	1.86	0.05	1.77 – 1.96	**<0.001**
Time	0.12	0.10	-0.08 – 0.31	0.240
$Condition_{Mindfulness}$	-0.06	0.12	-0.30 – 0.19	0.643
$Condition_{PMR}$	-0.01	0.12	-0.25 – 0.23	0.953
ISI_{2000}	0.15	0.12	-0.09 – 0.39	0.227
ISI_{4000}	0.39	0.12	0.15 – 0.63	**0.001**
$Age_{Grand\ Mean\ Centered}$	0.01	0.01	-0.01 – 0.02	0.347
Time : $Condition_{Mindfulness}$	0.30	0.25	-0.19 – 0.78	0.23
Time : $Condition_{PMR}$	0.62	0.24	0.15 – 1.09	**0.01**
Time : ISI_{2000}	0.13	0.24	-0.35 – 0.61	0.596
Time : ISI_{4000}	0.12	0.24	-0.36 – 0.60	0.616
$Condition_{Mindfulness}$: ISI_{2000}	0.23	0.30	-0.37 – 0.82	0.459
$Condition_{Mindfulness}$: ISI_{4000}	0.23	0.30	-0.37 – 0.82	0.459
$Condition_{PMR}$: ISI_{2000}	0.13	0.29	-0.44 – 0.71	0.653
$Condition_{PMR}$: ISI_{4000}	0.06	0.29	-0.52 – 0.63	0.845
Time : $Condition_{Mindfulness}$: ISI_{2000}	-0.65	0.61	-1.85 – 0.54	0.283
Time : $Condition_{Mindfulness}$: ISI_{4000}	-0.54	0.61	-1.73 – 0.66	0.378
Time : $Condition_{PMR}$: ISI_{2000}	-0.66	0.58	-1.81 – 0.49	0.260
Time : $Condition_{PMR}$: ISI_{4000}	-0.30	0.58	-1.44 – 0.85	0.614
Model Specifications				
Observations	462			
Df	443			
Deviance	499.889			
AIC	1387.515			
log-Likelihood	-673.757			

Note. P-values for fixed effects were calculated using Satterthwaites approximations. Confidence Intervals have been calculated using the Wald method.

Table C3

Linear Regression of Errors of Omission During the Continuous Performance Task

Predictors	*Estimates*	*SE*	*95% CI*	*p*
(Intercept)	6.41	0.57	5.29 – 7.53	**<0.001**
Time	0.17	1.14	-2.07 – 2.42	0.879
$Condition_{Mindfulness}$	-0.03	1.43	-2.84 – 2.78	0.983
$Condition_{PMR}$	-1.30	1.42	-4.09 – 1.48	0.359
ISI_{2000}	-4.96	1.40	-7.70 – -2.21	**<0.001**
ISI_{4000}	-8.14	1.40	-10.89 – -5.40	**<0.001**
$Age_{Grand\ Mean\ Centered}$	0.24	0.06	0.11 – 0.36	**<0.001**
Time : $Condition_{Mindfulness}$	-0.04	2.85	-5.65 – 5.57	0.989
Time : $Condition_{PMR}$	-6.64	2.75	-12.05 – -1.23	**0.016**
Time : ISI_{2000}	-2.42	2.79	-7.91 – 3.08	0.388
Time : ISI_{4000}	-3.38	2.79	-8.87 – 2.11	0.227
$Condition_{Mindfulness}$: ISI_{2000}	0.28	3.50	-6.59 – 7.15	0.936
$Condition_{Mindfulness}$: ISI_{4000}	-0.21	3.50	-7.08 – 6.66	0.952
$Condition_{PMR}$: ISI_{2000}	2.00	3.37	-4.61 – 8.62	0.552
$Condition_{PMR}$: ISI_{4000}	3.08	3.37	-3.54 – 9.70	0.361
Time : $Condition_{Mindfulness}$: ISI_{2000}	1.28	6.99	-12.46 – 15.02	0.855
Time : $Condition_{Mindfulness}$: ISI_{4000}	-0.92	6.99	-14.66 – 12.82	0.896
Time : $Condition_{PMR}$: ISI_{2000}	4.19	6.73	-9.05 – 17.42	0.534
Time : $Condition_{PMR}$: ISI_{4000}	2.90	6.73	-10.34 – 16.13	0.667
Model Specifications				
Observations	462			
Df	443			
Deviance	66289.447			
AIC	3645.493			
log-Likelihood	-1802.747			

Note. P-values for fixed effects were calculated using Satterthwaites approximations. Confidence Intervals have been calculated using the Wald method.

Table C4

Generalized Linear Mixed Model of RT During the N-Back Task

Predictors	*Estimates*	*SE*	*95% CI*	*p*
(Intercept)	531.47	5.23	521.22 – 541.72	**<0.001**
Time	-74.73	3.51	-81.61 – -67.84	**<0.001**
$Condition_{Mindfulness}$	1.12	3.80	-6.33 – 8.57	0.769
$Condition_{PMR}$	-29.48	4.35	-38.01 – -20.95	**<0.001**
$Nback_{2back}$	68.47	1.81	64.92 – 72.02	**<0.001**
$Nback_{3back}$	85.23	1.84	81.63 – 88.83	**<0.001**
$Age_{Grand\ Mean\ Centered}$	1.43	0.76	-0.06 – 2.92	0.059
Accuracy	-9.41	2.59	-14.48 – -4.34	**<0.001**
Target	8.88	1.24	6.45 – 11.31	**<0.001**
Time : $Condition_{Mindfulness}$	-35.98	6.70	-49.10 – -22.85	**<0.001**
Time : $Condition_{PMR}$	-21.24	5.02	-31.08 – -11.40	**<0.001**
Time : $Nback_{2back}$	-27.38	2.58	-32.44 – -22.32	**<0.001**
Time : $Nback_{3back}$	-10.85	2.77	-16.27 – -5.43	**<0.001**
$Condition_{Mindfulness}$: $Nback_{2back}$	-18.43	2.55	-23.44 – -13.43	**<0.001**
$Condition_{Mindfulness}$: $Nback_{3back}$	-8.91	2.65	-23.44 – -13.43	**<0.001**
$Condition_{PMR}$: $Nback_{2back}$	-25.08	2.97	-30.89 – -19.26	**<0.001**
$Condition_{PMR}$: $Nback_{3back}$	-14.30	2.93	-20.05 – -8.56	**<0.001**
Time : $Condition_{Mindfulness}$: $Nback_{2back}$	4.14	3.49	-2.70 – 10.97	0.235
Time : $Condition_{Mindfulness}$: $Nback_{3back}$	0.78	3.66	-6.39 – 7.95	0.832
Time : $Condition_{PMR}$: $Nback_{2back}$	-4.48	3.11	-10.58 – 1.62	0.150
Time : $Condition_{PMR}$: $Nback_{3back}$	3.66	4.33	-4.83 – 12.14	0.398

Random Effects		*Model Specifications*	
τ_{00} Subject	1165.75	Observations	41084
τ_{11} Subject : Time	1106.84	Df	41059
ρ_{01} Subject	-0.17	Deviance	532840.305
N Subject	78	AIC	532890.305
		log-Likelihood	-266420.152

Note. P-values for fixed effects were calculated using Satterthwaites approximations. Confidence Intervals have been calculated using the Wald method. Model fit by maximum likelihood (Laplace Approximation); family: inverse.gaussian (identity).

Table C5

Linear Regression of d' During the N-Back Task

Predictors	*Estimates*	*SE*	*95% CI*	*p*
(Intercept)	2.26	0.05	2.17 – 2.35	**<0.001**
Time	0.02	0.09	-0.16 – 0.20	0.820
$Condition_{Mindfulness}$	0.16	0.12	-0.07 – 0.39	0.167
$Condition_{PMR}$	0.12	0.12	-0.11 – 0.35	0.292
$Nback_{2back}$	-0.48	0.11	-0.70 – -0.27	**<0.001**
$Nback_{3back}$	-1.30	0.11	-1.52 – -1.08	**<0.001**
$Age_{Grand\ Mean\ Centered}$	-0.01	0.00	-0.01 – 0.00	0.183
Time : $Condition_{Mindfulness}$	0.12	0.22	-0.30 – 0.55	0.568
Time : $Condition_{PMR}$	0.21	0.22	-0.22 – 0.64	0.333
Time : $Nback_{2back}$	0.33	0.22	-0.10 – 0.77	0.132
Time : $Nback_{3back}$	0.27	0.22	-0.17 – 0.70	0.231
$Condition_{Mindfulness}$: $Nback_{2back}$	-0.06	0.27	-0.59 – 0.46	0.814
$Condition_{Mindfulness}$: $Nback_{3back}$	-0.16	0.27	-0.68 – 0.37	0.560
$Condition_{PMR}$: $Nback_{2back}$	-0.17	0.27	-0.70 – 0.36	0.525
$Condition_{PMR}$: $Nback_{3back}$	-0.22	0.27	-0.75 – 0.31	0.412
Time : $Condition_{Mindfulness}$: $Nback_{2back}$	-0.62	0.53	-1.67 – 0.43	0.245
Time : $Condition_{Mindfulness}$: $Nback_{3back}$	-0.76	0.53	-1.81 – 0.29	0.155
Time : $Condition_{PMR}$: $Nback_{2back}$	-0.26	0.54	-1.31 – 0.79	0.625
Time : $Condition_{PMR}$: $Nback_{3back}$	-0.18	0.54	-1.24 – 0.89	0.746
Model Specifications				
Observations	495			
Df	476			
Deviance	476.863			
AIC	1426.271			
log-Likelihood	-693.136			

Note. P-values for fixed effects were calculated using Satterthwaites approximations. Confidence Intervals have been calculated using the Wald method.

Table C6

Linear Regression of Errors of Omission During the N-Back Task

Predictors	*Estimates*	*SE*	*95% CI*	*p*
(Intercept)	6.51	0.21	6.10 – 6.92	**<0.001**
Time	0.15	0.21	-0.26 – 0.56	0.469
$Condition_{Mindfulness}$	-0.59	0.54	-1.64 – 0.46	0.271
$Condition_{PMR}$	-0.17	0.54	-1.23 – 0.90	0.758
$Nback_{2back}$	1.05	0.52	0.04 – 2.06	**0.043**
$Nback_{3back}$	5.54	0.52	4.52 – 6.55	**<0.001**
$Age_{Grand\ Mean\ Centered}$	0.03	0.02	-0.01 – 0.07	0.179
Time : $Condition_{Mindfulness}$	-0.66	0.51	-1.66 – 0.34	0.194
Time : $Condition_{PMR}$	-1.03	0.51	-2.03 – -0.03	**0.044**
Time : $Nback_{2back}$	0.46	0.52	-0.55 – 1.48	0.369
Time : $Nback_{3back}$	0.47	0.52	-0.54 – 1.48	0.362
$Condition_{Mindfulness}$: $Nback_{2back}$	0.33	1.24	-2.11 – 2.77	0.788
$Condition_{Mindfulness}$: $Nback_{3back}$	-1.09	1.24	-3.53 – 1.35	0.382
$Condition_{PMR}$: $Nback_{2back}$	-0.24	1.24	-2.69 – 2.20	0.844
$Condition_{PMR}$: $Nback_{3back}$	-0.41	1.25	-2.87 – 2.06	0.744
Time : $Condition_{Mindfulness}$: $Nback_{2back}$	-0.51	1.24	-2.94 – 1.93	0.684
Time : $Condition_{Mindfulness}$: $Nback_{3back}$	1.53	1.24	-0.91 – 3.97	0.218
Time : $Condition_{PMR}$: $Nback_{2back}$	0.45	1.24	-2.00 – 2.89	0.720
Time : $Condition_{PMR}$: $Nback_{3back}$	0.47	1.25	-2.00 – 2.93	0.709
Model Specifications				
Observations	462			
Df	443			
Deviance	66289.447			
AIC	3645.493			
log-Likelihood	-1802.747			

Note. P-values for fixed effects were calculated using Satterthwaites approximations. Confidence Intervals have been calculated using the Wald method.

Table C7

Generalized Linear Mixed Model of RT During the Number-Letter Task

Predictors	*Estimates*	*SE*	*95% CI*	*p*
(Intercept)	583.70	5.84	572.26 – 595.14	**<0.001**
Time	-80.91	4.79	-90.30 – -71.52	**<0.001**
$Condition_{Mindfulness}$	2.77	6.19	-9.36 – 14.91	0.654
$Condition_{PMR}$	4.64	5.43	-6.00 – 15.28	0.393
Switch	97.76	3.59	90.74 – 104.79	**<0.001**
Accuracy	26.12	5.54	15.25 – 36.98	**<0.001**
$Age_{Grand\ Mean\ Centered}$	1.42	0.77	-0.09 – 2.94	0.066
Time : $Condition_{Mindfulness}$	-28.08	6.78	-41.36 – -14.79	**<0.001**
Time : $Condition_{PMR}$	-9.90	5.89	-21.44 – 1.63	0.092
Time : Switch	-44.77	5.02	-54.61 – -34.92	**<0.001**
$Condition_{Mindfulness}$: Switch	-39.44	4.67	-48.59 – -30.29	**<0.001**
$Condition_{PMR}$: Switch	-18.97	5.55	-29.85 – -8.10	**0.001**
Time : $Condition_{Mindfulness}$: Switch	-2.76	6.68	-15.85 – 10.33	0.679
Time : $Condition_{PMR}$: Switch	29.4	7.73	14.24 – 44.56	**<0.001**

Random Effects		*Model Specifications*	
τ_{00} Subject	1873.85	Observations	20980
τ_{11} Subject : Time	1092.14	Df	20962
ρ_{01} Subject	-0.08	Deviance	293658.086
N Subject	77	AIC	293694.086
		log-Likelihood	-146829.043

Note. P-values for fixed effects were calculated using Satterthwaites approximations. Confidence Intervals have been calculated using the Wald method. Model fit by maximum likelihood (Laplace Approximation); family: inverse.gaussian (identity).

Table C8

Multilevel Logistic Regression of Accuracy During the Number-Letter Task

Predictors	*Estimates*	*SE*	*95% CI*	*p*
(Intercept)	2.90	0.09	2.72 – 3.08	**<0.001**
Switch	-0.33	0.06	-0.45 – -0.22	**<0.001**
$\text{Condition}_{\text{Mindfulness}}$	-0.49	0.23	-0.93 – -0.05	**0.030**
$\text{Condition}_{\text{PMR}}$	-0.11	0.23	-0.55 – 0.34	0.639
Time	-0.05	0.09	-0.21 – 0.12	0.583
$\text{Age}_{\text{Grand Mean Centered}}$	-0.01	0.01	-0.02 – 0.01	0.404
$\text{Condition}_{\text{Mindfulness}}$: Switch	0.20	0.14	-0.07 – 0.48	0.152
$\text{Condition}_{\text{PMR}}$: Switch	0.19	0.15	-0.10 – 0.49	0.200
Time : Switch	-0.26	0.12	-0.50 – -0.03	**0.027**
Time : $\text{Condition}_{\text{Mindfulness}}$	0.09	0.20	-0.30 – 0.48	0.667
Time : $\text{Condition}_{\text{PMR}}$	0.10	0.21	-0.31 – 0.50	0.638
Time : $\text{Condition}_{\text{Mindfulness}}$: Switch	0.35	0.28	-0.21 – 0.90	0.219
Time : $\text{Condition}_{\text{PMR}}$: Switch	0.18	0.30	-0.41 – 0.77	0.553

Random Effects		*Model Specifications*	
τ_{00} Subject	0.55	Observations	20980
τ_{11} Subject : Time	0.20	Df	20964
ρ_{01} Subject	-0.10	Deviance	9216.621
N Subject	77	AIC	9248.621
		log-Likelihood	-4608.311

Note. P-values for fixed effects were calculated using Satterthwaites approximations. Confidence Intervals have been calculated using the Wald method. Model fit by maximum likelihood (Laplace Approximation); family: binomial (logit).

Table C9

Generalized Linear Mixed Model of RT During the Attention Network Task for the Executive Control Network

Predictors	*Estimates*	*SE*	*95% CI*	*p*
(Intercept)	375.98	2.74	370.62 – 381.35	**<0.001**
Target	103.58	0.90	101.82 – 105.35	**<0.001**
$\text{Age}_{\text{Grand Mean Centered}}$	3.39	0.53	2.35 – 4.43	**<0.001**
Accuracy	82.79	1.32	80.20 – 85.37	**<0.001**
Time	-37.82	2.55	-42.81 – -32.82	**<0.001**
$\text{Condition}_{\text{Mindfulness}}$	-20.7	3.72	-28.00 – -13.41	**<0.001**
$\text{Condition}_{\text{PMR}}$	13.02	3.68	5.82 – 20.23	**<0.001**
$\text{Cue}_{\text{Middle}}$	-11.29	1.10	-13.45 – -9.14	**<0.001**
$\text{Cue}_{\text{Spatial}}$	-12.32	1.11	-14.49 – -10.14	**<0.001**
$\text{Cue}_{\text{Double}}$	-11.93	1.10	-14.09 – -9.77	**<0.001**
Time : $\text{Condition}_{\text{Mindfulness}}$	0.07	4.51	-8.77 – 8.92	0.987
Time : $\text{Condition}_{\text{PMR}}$	-7.33	4.12	-15.40 – 0.74	0.075
Time : Target	-21.44	1.66	-24.70 – -18.18	**<0.001**
$\text{Condition}_{\text{Mindfulness}}$: Target	-10.18	1.84	-13.80 – -6.57	**<0.001**
$\text{Condition}_{\text{PMR}}$: Target	3.60	2.06	-0.44 – 7.63	0.081
Time : $\text{Condition}_{\text{Mindfulness}}$: Target	-12.66	2.58	-17.72 – -7.61	**<0.001**
Time : $\text{Condition}_{\text{PMR}}$: Target	-14.02	2.57	-19.05 – -8.99	**<0.001**

Random Effects		*Model Specifications*	
τ_{00} Subject	261.62	Observations	46658
τ_{11} Subject : Time	354.24	Df	46637
ρ_{01} Subject	-0.07	Deviance	558291.759
N Subject	77	AIC	558333.759
		log-Likelihood	-279145.88

Note. P-values for fixed effects were calculated using Satterthwaites approximations. Confidence Intervals have been calculated using the Wald method. Model fit by maximum likelihood (Laplace Approximation); family: inverse.gaussian (identity).

Table C10

Generalized Linear Mixed Model of RT During the Attention Network Task for the Alerting & Orienting Network

Predictors	*Estimates*	*SE*	*95% CI*	*p*
(Intercept)	374.36	3.76	367.00 – 381.73	**<0.001**
Target	101.11	0.92	99.30 – 102.91	**<0.001**
$Age_{Grand\ Mean\ Centered}$	3.41	0.55	2.33 – 4.49	**<0.001**
Accuracy	84.13	1.34	81.50 – 86.76	**<0.001**
Time	-34.76	3.80	-42.20 – -27.31	**<0.001**
$Condition_{Mindfulness}$	-19.06	5.48	-29.80 – -8.33	**0.001**
$Condition_{PMR}$	12.41	8.37	-4.00 – 28.82	0.138
Cue_{Middle}	-11.39	1.17	-13.69 – -9.09	**<0.001**
$Cue_{Spatial}$	-12.67	1.16	-14.94 – -10.40	**<0.001**
Cue_{Double}	-11.85	1.18	-14.17 – -9.53	**<0.001**
Time : $Condition_{Mindfulness}$	1.97	7.30	-12.34 – 16.28	0.787
Time : $Condition_{PMR}$	-5.34	4.64	-14.43 – 3.76	0.250
Time : Cue_{Middle}	1.86	1.98	-2.02 – 5.74	0.347
Time : $Cue_{Spatial}$	1.32	1.97	-2.55 – 5.19	0.504
Time : Cue_{Double}	-2.71	1.93	-6.49 – 1.07	0.160
$Condition_{Mindfulness}$: Cue_{Middle}	2.65	2.30	-1.86 – 7.15	0.250
$Condition_{Mindfulness}$: $Cue_{Spatial}$	1.89	2.58	-3.16 – 6.94	0.463
$Condition_{Mindfulness}$: Cue_{Double}	3.56	2.21	-0.77 – 7.89	0.107
$Condition_{PMR}$: Cue_{Middle}	6.33	2.65	1.13 – 11.52	**0.017**
$Condition_{PMR}$: $Cue_{Spatial}$	-1.95	2.75	-7.33 – 3.43	0.478
$Condition_{PMR}$: Cue_{Double}	4.62	2.76	-0.80 – 10.04	0.095
Time : $Condition_{Mindfulness}$: Cue_{Middle}	12.36	3.49	5.52 – 19.20	**<0.001**
Time : $Condition_{Mindfulness}$: $Cue_{Spatial}$	9.23	3.49	2.39 – 16.07	**0.008**
Time : $Condition_{Mindfulness}$: Cue_{Double}	3.51	4.27	-4.87 – 11.88	0.412
Time : $Condition_{PMR}$: Cue_{Middle}	6.00	3.76	-1.37 – 13.37	0.110
Time : $Condition_{PMR}$: $Cue_{Spatial}$	12.49	5.09	2.52 – 22.46	**0.014**
Time : $Condition_{PMR}$: Cue_{Double}	9.54	4.39	0.94 – 18.13	**0.030**

Random Effects		*Model Specifications*	
τ_{00} Subject	262.41	Observations	46658
τ_{11} Subject : Time	353.58	Df	46627
ρ_{01} Subject	-0.08	Deviance	558476.054
N Subject	77	AIC	558538.054
		log-Likelihood	-279238.027

Note. P-values for fixed effects were calculated using Satterthwaites approximations. Confidence Intervals have been calculated using the Wald method. Model fit by maximum likelihood (Laplace Approximation); family: inverse.gaussian (identity).

Table C11

Multilevel Logistic Regression of Accuracy During the Attention Network Task for the Executive Control Network

Predictors	*Estimates*	*SE*	*95% CI*	*p*
(Intercept)	3.73	0.14	3.45 – 4.01	**<0.001**
Target	-2.76	0.07	-2.89 – -2.64	**<0.001**
$Condition_{Mindfulness}$	0.40	0.36	-0.30 – 1.10	0.265
$Condition_{PMR}$	0.22	0.37	-0.51 – 0.95	0.558
Time	0.03	0.14	-0.24 – 0.30	0.838
Cue_{Middle}	-0.08	0.06	-0.19 – 0.04	0.193
$Cue_{Spatial}$	-0.09	0.06	-0.21 – 0.03	0.132
Cue_{Double}	-0.07	0.06	-0.19 – 0.04	0.211
$Age_{Grand\ Mean\ Centered}$	0.05	0.02	0.02 – 0.08	**0.001**
$Condition_{Mindfulness}$: Target	-2.06	0.16	-2.38 – -1.74	**<0.001**
$Condition_{PMR}$: Target	-0.31	0.15	-0.60 – -0.01	**0.042**
Time : Target	0.27	0.13	0.02 – 0.53	**0.034**
Time : $Condition_{Mindfulness}$	-0.26	0.33	-0.90 – 0.39	0.437
Time : $Condition_{PMR}$	-0.49	0.34	-1.15 – 0.16	0.141
Time : $Condition_{Mindfulness}$: Target	0.13	0.33	-0.52 – 0.77	0.693
Time : $Condition_{PMR}$: Target	0.02	0.30	-0.57 – 0.61	0.946

Random Effects		*Model Specifications*	
τ_{00} Subject	1.48	Observations	46843
τ_{11} Subject : Time	1.00	Df	46824
ρ_{01} Subject	-0.25	Deviance	16872.924
N Subject	77	AIC	16910.924
		log-Likelihood	-8436.462

Note. P-values for fixed effects were calculated using Satterthwaites approximations. Confidence Intervals have been calculated using the Wald method. Model fit by maximum likelihood (Laplace Approximation); family: binomial (logit).

Table C12

Multilevel Logistic Regression of Accuracy During the Attention Network Task for the Alerting & Orienting Network

Predictors	*Estimates*	*SE*	*95% CI*	*p*
(Intercept)	3.78	0.14	3.50 – 4.06	**<0.001**
Cue_{Middle}	-0.08	0.06	-0.19 – 0.04	0.210
$Cue_{Spatial}$	-0.09	0.06	-0.20 – 0.03	0.149
Cue_{Double}	-0.06	0.06	-0.18 – 0.06	0.340
$Condition_{Mindfulness}$	-0.42	0.34	-1.09 – 0.25	0.218
$Condition_{PMR}$	0.11	0.36	-0.59 – 0.81	0.761
Time	0.14	0.14	-0.12 – 0.41	0.298
Target	-2.90	0.07	-3.03 – -2.77	**<0.001**
$Age_{Grand\ Mean\ Centered}$	0.05	0.02	0.02 – 0.08	**0.001**
$Condition_{Mindfulness}$: Cue_{Middle}	-0.04	0.14	-0.33 – 0.24	0.771
$Condition_{Mindfulness}$: $Cue_{Spatial}$	-0.09	0.14	-0.37 – 0.20	0.552
$Condition_{Mindfulness}$: Cue_{Double}	-0.16	0.14	-0.45 – 0.12	0.252
$Condition_{PMR}$: Cue_{Middle}	-0.11	0.15	-0.42 – 0.19	0.459
$Condition_{PMR}$: $Cue_{Spatial}$	-0.02	0.15	-0.32 – 0.28	0.886
$Condition_{PMR}$: Cue_{Double}	-0.01	0.16	-0.31 – 0.30	0.972
Time : Cue_{Middle}	0.15	0.12	-0.08 – 0.39	0.206
Time : $Cue_{Spatial}$	-0.03	0.12	-0.26 – 0.20	0.802
Time : Cue_{Double}	-0.05	0.12	-0.29 – 0.18	0.649
Time : $Condition_{Mindfulness}$	-0.21	0.32	-0.84 – 0.41	0.504
Time : $Condition_{PMR}$	-0.53	0.34	-1.19 – 0.13	0.118
Time : $Condition_{Mindfulness}$: Cue_{Middle}	-0.40	0.29	-0.96 – 0.17	0.167
Time : $Condition_{Mindfulness}$: $Cue_{Spatial}$	-0.28	0.28	-0.84 – 0.27	0.318
Time : $Condition_{Mindfulness}$: Cue_{Double}	-0.33	0.29	-0.89 – 0.23	0.251
Time : $Condition_{PMR}$: Cue_{Middle}	0.06	0.31	-0.54 – 0.67	0.840
Time : $Condition_{PMR}$: $Cue_{Spatial}$	0.27	0.31	-0.33 – 0.87	0.380
Time : $Condition_{PMR}$: Cue_{Double}	-0.19	0.31	-0.80 – 0.42	0.546

Random Effects		*Model Specifications*	
τ_{00} Subject	1.40	Observations	46843
τ_{11} Subject : Time	1.14	Df	46814
ρ_{01} Subject	-0.28	Deviance	17056.148
N Subject	77	AIC	17114.148
		log-Likelihood	-8528.074

Note. P-values for fixed effects were calculated using Satterthwaites approximations. Confidence Intervals have been calculated using the Wald method. Model fit by maximum likelihood (Laplace Approximation); family: binomial (logit).
