# Supplementary material for: Mechanisms Underlying Cognitive Effects of Inducing a Mindful State
Source: J Cogn. 2022 Jan 21;5(1):13. doi: 10.5334/joc.205 (PMC9400668; doi:10.5334/joc.205)
Supplement: Appendix D. — Generalized Linear Mixed Model Comparison of Measures of Attentional Control and Executive Functioning. [file joc-5-1-205-s4.pdf]

Appendix D

Generalized Linear Mixed Model Comparison of Measures of Attentional Control and Executive Functioning

							LRT Test against final		
Task	*Model*	*AIC*	*BIC*	*logLik*	*deviance*	*df*	*Chi df*	*X2*	*p*
CPT	Interaction (final)	536187	536389	-268071	536141	23	-	-	-
	Main Effects	536273	536370	-268126	536251	11	12	110.13	**< 0.001**
	Random Effects	539286	539330	-269638	539276	5	18	3134.60	**< 0.001**
N-Back	Interaction (final)	532890	533106	-266420	532840	25	-	-	-
	Main Effects	532952	533064	-266463	532926	13	12	85.76	**<0.001**
	Random Effects	535108	535151	-267549	535098	5	20	2257.90	**<0.001**
Number-Letter	Interaction (final)	293694	293837	-146829	293658	18	-	-	-
	Main Effects	293727	293814	-146853	293705	11	7	46.95	**<0.001**
	Random Effects	294254	294293	-147122	294244	5	13	585.47	**<0.001**
ANT Executive Network	Interaction (final)	558334	558518	-279146	558292	21	-	-	-
	Main Effects	558532	558654	-279252	558504	14	7	212.05	**< 0.001**
	Random Effects	570127	570170	-285058	570117	5	16	11825	**< 0.001**
ANT Alerting & Orienting Network	Interaction (final)	558538	558809	-279238	558476	31	-	-	-
	Main Effects	558532	558654	-279252	558504	14	17	27.75	**0.048**
	Random Effects	570127	570170	-285058	570117	5	26	11641	**< 0.001**

Note . LTR = Likelihood ratio tests. Comparisons against models with main effects only and random effects only
